# Transcriptional regulatory networks of tumor-associated macrophages that drive malignancy in mesenchymal glioblastoma

**DOI:** 10.1186/s13059-020-02140-x

**Published:** 2020-08-26

**Authors:** Jason K. Sa, Nakho Chang, Hye Won Lee, Hee Jin Cho, Michele Ceccarelli, Luigi Cerulo, Jinlong Yin, Sung Soo Kim, Francesca P. Caruso, Mijeong Lee, Donggeon Kim, Young Taek Oh, Yeri Lee, Nam-Gu Her, Byeongkwi Min, Hye-Jin Kim, Da Eun Jeong, Hye-Mi Kim, Hyunho Kim, Seok Chung, Hyun Goo Woo, Jeongwu Lee, Doo-Sik Kong, Ho Jun Seol, Jung-Il Lee, Jinho Kim, Woong-Yang Park, Qianghu Wang, Erik P. Sulman, Amy B. Heimberger, Michael Lim, Jong Bae Park, Antonio Iavarone, Roel G. W. Verhaak, Do-Hyun Nam

**Affiliations:** 1 Department of Biomedical Sciences, Korea University College of Medicine, Seoul, South Korea; 2 Yuhan Research Institute, Yongin, South Korea; 3 Department of Hospital Medicine, Yonsei University College of Medicine, Seoul, South Korea; 4 Innovative Therapeutic Research Center, Precision Medicine Research Institute, Samsung Medical Center, Seoul, South Korea; 5 Department of Electrical Engineering and Information Technology (DIETI), University of Naples “Federico II”, Naples, Italy; 6 Biogem, Instituto di Biologia e Genetica Molecolare, Ariano Irpino, Italy; 7 Department of Science and Technology, University of Sannio, Benevento, Italy; 8 Henan and Macquarie University Joint Centre for Biomedical Innovation, School of Life Sciences, Henan University, Kaifeng, Henan China; 9 Department of Cancer Biomedical Science, Graduate School of Cancer Science and Policy, National Cancer Center, Goyang, South Korea; 10 Rare Cancer Branch, Research Institute and Hospital, National Cancer Center, Goyang, South Korea; 11 Biogem Scarl, Instituto di Ricerche Genetiche “Gaetano Salvatore”, Ariano Irpino, Italy; 12 Institute for Refractory Cancer Research, Samsung Medical Center, Seoul, South Korea; 13 Institute for Cancer Genetics, Columbia University, New York, NY USA; 14 AIMEDBIO Inc., Seoul, South Korea; 15 Department of Health Science & Technology, Samsung Advanced Institute for Health Sciences & Technology, Sungkyunkwan University, Seoul, South Korea; 16 Department of Anatomy and Cell Biology, Sungkyunkwan University School of Medicine, Suwon, South Korea; 17 School of Mechanical Engineering, Korea University, Seoul, South Korea; 18 Department of Physiology, Ajou University School of Medicine, Suwon, South Korea; 19 Graduate School of Biomedical Science, Ajou University School of Medicine, Suwon, South Korea; 20 Department of Cancer Biology, Lerner Research Institute, Cleveland Clinic, Cleveland, OH USA; 21 Department of Neurosurgery, Samsung Medical Center, Sungkyunkwan University School of Medicine, Seoul, South Korea; 22 Samsung Genome Institute, Samsung Medical Center, Seoul, South Korea; 23 Department of Bioinformatics, School of Biomedical Engineering and Informatics, Nanjing Medical University, Nanjing, China; 24 Department of Radiation Oncology, NYU Grossman School of Medicine, New York, NY USA; 25 Department of Neurosurgery, University of Texas MD Anderson Cancer Center, Houston, TX USA; 26 Department of Neurosurgery, Johns Hopkins University School of Medicine, Baltimore, MD USA; 27 Department of Pathology, Columbia University, New York, NY USA; 28 Department of Neurology, Columbia University, New York, NY USA; 29 The Jackson Laboratory for Genomic Medicine, Farmington, CT USA

## Abstract

**Background:**

Glioblastoma (GBM) is a complex disease with extensive molecular and transcriptional heterogeneity. GBM can be subcategorized into four distinct subtypes; tumors that shift towards the mesenchymal phenotype upon recurrence are generally associated with treatment resistance, unfavorable prognosis, and the infiltration of pro-tumorigenic macrophages.

**Results:**

We explore the transcriptional regulatory networks of mesenchymal-associated tumor-associated macrophages (MA-TAMs), which drive the malignant phenotypic state of GBM, and identify macrophage receptor with collagenous structure (MARCO) as the most highly differentially expressed gene. MARCO^high^ TAMs induce a phenotypic shift towards mesenchymal cellular state of glioma stem cells, promoting both invasive and proliferative activities, as well as therapeutic resistance to irradiation. MARCO^high^ TAMs also significantly accelerate tumor engraftment and growth in vivo. Moreover, both MA-TAM master regulators and their target genes are significantly correlated with poor clinical outcomes and are often associated with genomic aberrations in neurofibromin 1 (NF1) and phosphoinositide 3-kinases/mammalian target of rapamycin/Akt pathway (PI3K-mTOR-AKT)-related genes. We further demonstrate the origination of MA-TAMs from peripheral blood, as well as their potential association with tumor-induced polarization states and immunosuppressive environments.

**Conclusions:**

Collectively, our study characterizes the global transcriptional profile of TAMs driving mesenchymal GBM pathogenesis, providing potential therapeutic targets for improving the effectiveness of GBM immunotherapy.

## Introduction

Glioblastoma (GBM) is the most common and lethal primary brain tumor in adults [1]. The current standard treatment regimen primarily provides palliative treatment and leads to a median survival of less than 15 months despite aggressive therapeutic interventions, including maximum surgical resection followed by chemo- and radiotherapy [2]. GBMs can be subcategorized into distinct subtypes based on their transcriptional cellular state and accompanying unique genomic alterations [3, 4]. This expression-based classification system has emerged as an important concept in understanding the biological behavior and genomic complexity of GBM. Its significance has been increasingly recognized owing to the distinct clinical response of each subtype to current treatment strategies, their diverse cellular origins and differentiation hierarchies, and the infiltration and accumulation of tumor-associated immune cells within the surrounding environment [4–8].

Tumor-associated macrophages (TAMs) within the GBM niche include a mixture of heterogeneous subpopulations that are derived from two major sources: brain-resident microglial cells and peripheral blood-derived monocytes [9–11]. These macrophages present specific cell-surface antigens and play distinct functional roles based on their cellular origin and tumor-induced polarization state [12, 13]. M1-polarized macrophages have been recognized for their potent anti-tumor immune cytotoxicity function, while M2 macrophages promote tumor progression through modulation of immune-suppressive environment and pro-tumorigenic functions [14]. Notably, GBMs demonstrate a high degree of subtype plasticity, manifesting dynamic subtype transitions between diagnosis and recurrence [4, 6, 15]. In particular, mesenchymal transformation has been generally associated with limited clinical response to current standard therapies, largely due to the infiltration of pro-tumorigenic immune cells such as TAMs [16–18].

GBM contains a subpopulation of highly tumorigenic stem-like cells known as glioma stem cells (GSCs); these cells possess an innate perpetual self-renewal and differentiation ability [19, 20]. While previous studies have identified molecular links between GSCs and tumor microenvironment (TME) that promote recruitment of TAMs within the perivascular region [21–25], the major transcriptional regulatory networks of TAMs driving the malignant phenotypic state of mesenchymal GBM still remain obscure. To address such challenges, we performed comprehensive transcriptome analyses of mesenchymal-associated tumor-associated macrophages (MA-TAMs) and identified its unique signature and core master regulators that govern their transcriptional cellular state. Furthermore, MA-TAMs promoted mesenchymal trans-differentiation of GSCs in vitro and in vivo and confer unfavorable prognosis in GBM patients. Collectively, our results unraveled the dynamic transcriptomic networks of MA-TAMs that govern mesenchymal transition of GSCs and present potential immunotherapeutic targets that can be exploited in the field of GBM treatment.

## Results

### Identification of MA-TAM-encoding genes and its transcriptional master regulators

**Fig. 1:**
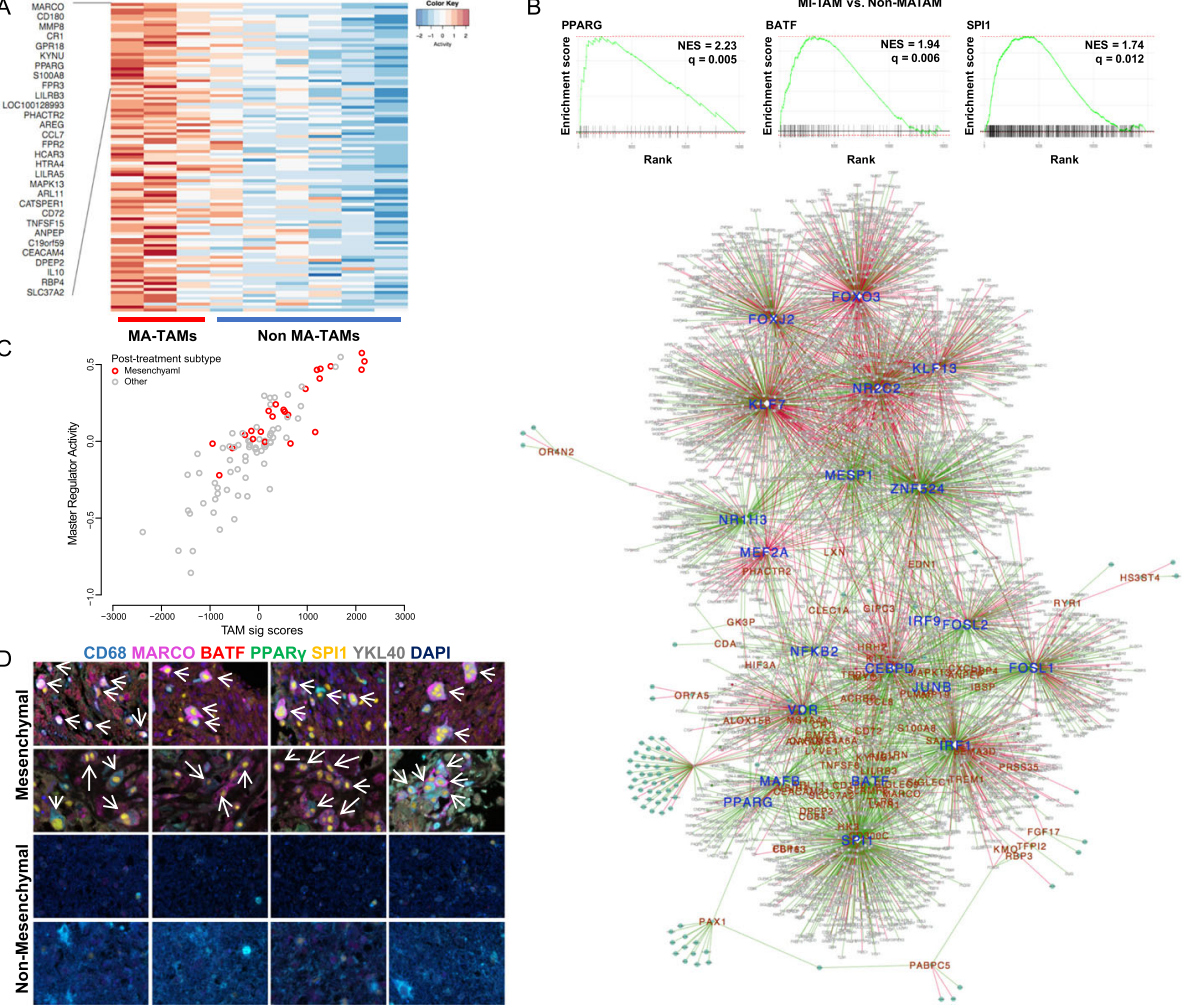
Identification of transcriptional regulatory networks of mesenchymal-associated tumor-associated macrophages (MA-TAMs). **a** Heatmap representation of MA-TAM encoding genes in TAMs that were isolated from three mesenchymal (MA-TAMs) and six non-mesenchymal (non-MA-TAMs) tumor specimens. **b** Gene Set Enrichment Analysis (GSEA) of *PPARG*, *BATF*, and *SPI1* in MA-TAM compared to non-MA-TAM (upper panel). Global regulatory networks between MA-TAM master regulators (blue) and their target genes (red) (bottom panel). Positive and negative interactions between master regulators and their target genes are represented in green and red, respectively. Master regulators with *P* < 0.05 are highlighted in boldfaced blue. **c** Scatter plot correlation between MA-TAM signature score and master regulator activity in 91 longitudinal pair samples. Recurrent tumors were categorized into two groups: mesenchymal and non-mesenchymal. **d** Multiplex immunohistochemical analysis of glioblastoma specimens classified as either mesenchymal or non-mesenchymal. The co-localization of CD68, MARCO, BATF, PPARG, SPI1, and YKL40 are indicated with white arrows

To identify MA-TAM-specific gene signature and its transcriptional master regulators that potentially govern mesenchymal cellular differentiation of GSCs, TAM-enriched subpopulations were identified and isolated from human primary GBM specimens via fluorescence-activated cell sorting (FACS) and subjected to whole-transcriptome sequencing (WTS). Isolated TAMs were classified as either MA-TAMs or non-MA-TAMs based on subtype classification of the corresponding tumor cells [4]. We first identified MA-TAM-encoding genes by extracting a set of transcriptomes from isolated MA-TAMs that were highly correlated with mesenchymal activity of the corresponding tumor cells (Additional file 1: Figure S1). Thereafter, we selected candidate genes that were uniquely expressed by non-malignant cells using GBM orthotopic patient-derived xenografts (PDX) models [26], which we considered as *bona fide* tumor-associated stromal/immune cells. The resulting genes consisted of various key macrophage-associated molecules, including macrophage receptor with collagenous structure (*MARCO*), chemokine (C-C motif) ligand 7 (*CCL7*), and matrix metallopeptidase 8 (*MMP8*) and were significantly enriched for inflammatory response and activation of various immune-associated pathways (Fig. 1a, Additional file 1: Figure S2). Using the previously established regularized gradient boosting machine (RGBM) algorithm [27–29], we reconstructed glioma-specific global regulatory networks from 1250 mRNA profiles obtained from The Cancer Genome Atlas (TCGA) and applied them to our MA-TAM gene signature. As a result, we identified twenty-one candidate transcriptional master regulators, including peroxisome proliferator-activated receptor gamma (*PPARG*), Spi-1 proto-oncogene (*SPI1*), and basic leucine zipper ATF-like transcription factor (*BATF*) (Fig. 1b, Additional file 1: Figure S3).

Because recent large-scale longitudinal GBM studies have demonstrated a high degree of subtype plasticity during tumor evolutionary dynamics [4, 6, 30], we evaluated transcriptome shifts in MA-TAM encoding genes and their core regulators between diagnosis and relapse in ninety-one paired gliomas. Notably, we discovered significant correlations between increased levels of MA-TAM target genes and their master regulators with subtype transitions into the mesenchymal cellular state. (Fig. 1c). Additionally, multi-color immunohistochemistry (IHC) analyses of human GBM specimens demonstrated prevalence of putative MA-TAM master regulators and their target genes, including *MARCO*, in mesenchymal-classified tumors, further confirming the authenticity and feasibility of our systematic process to identify MA-TAM transcriptional networks (Fig. 1d and Additional file 1: Figure S4). Collectively, our results have identified potential key molecules of TAMs that promote the malignant state of mesenchymal GBM.

### MARCO^high^ TAMs drive mesenchymal phenotypic state of GSCs in vitro

**Fig. 2:**
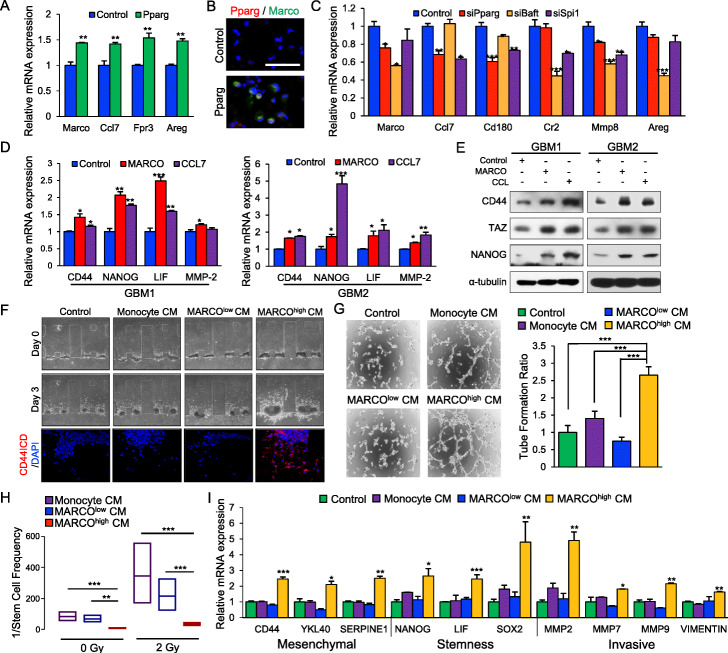
MARCO promotes mesenchymal, invasive, and migratory phenotypes, as well as therapeutic resistance to irradiation. **a** qRT-PCR analysis to determine the effects of *Pparg* on the mRNA expression levels of the MA-TAM genes *Marco*, *Ccl7*, *Fpr3*, and *Areg* in mouse peritoneal macrophages. **b** Representative immunofluorescence images of *Pparg* and *Marco* in mouse peritoneal macrophages transduced with either control or *Pparg.* **c** qRT-PCR analysis to determine the effects of si*PPARG*, si*Batf*, or si*Spi1* on the mRNA expression levels of the MA-TAM genes *Marco*, *Ccl7*, *Cd180*, *Cr2*, *Mmp8*, and *Areg* in mouse peritoneal macrophages. **d** qRT-PCR analysis to determine the effects of MARCO or CCL7 on the expression of mesenchymal, stemness, and cellular invasion markers including *CD44*, *NANOG*, *LIF*, and *MMP-2* in two GSC samples. **e** Immunoblot analysis of CD44, TAZ, and NANOG activity in GSCs treated with either control, MARCO, or CCL7; α-tubulin was used as a loading control. **f** Representative images of 3D invasion assays in GSCs treated with either control, monocyte-derived CM, MARCO^low^ TAM-derived CM, or MARCO^high^ TAM-derived CM (upper panel). Immunofluorescence images of CD44 intracellular domain and DAPI (red and blue, respectively; lower panel). **g** Representative images of tube formation assay (left panel) and representative bar graphs (right panel) for HUVECs. **h** The 1/stem cell frequency of GSCs treated with either monocyte-derived CM, MARCO^low^ TAM-derived CM, or MARCO^high^ TAM-derived CM and subjected to 2 Gy ionizing radiation. Stem cell frequency was calculated by extreme limiting dilution analysis. **i** qRT-PCR analysis to determine the effects of monocyte-derived CM, MARCO^low^ TAM-derived CM, and MARCO^high^ TAM-derived CM on mesenchymal, stemness, and cellular invasion markers in GSCs. *P ≤ 0.05, **P ≤ 0.01, ***P ≤ 0.001. Data shown in **a**, **c**, **d**, and **i** are representative of three independent and reproducible experiments. Data shown in **b** and **e**–**h** are representative of two independent and reproducible experiments

To assess whether MA-TAM master regulators govern the transcriptome expression levels of their target genes, we generated an overexpression construct of *Pparg* and transduced it into freshly harvested mouse peritoneal macrophages. Notably, overexpression of *Pparg* significantly increased the expression levels of MA-TAM-encoding genes, including *Marco*, *Ccl7*, formyl peptide receptor 3 (*Fpr3*), and Amphiregulin (*Areg*) (Fig. 2a, b). Conversely, disruption of *Pparg*, *Batf*, or *Spi1* expression via shRNA-mediated knockdown considerably attenuated their target gene expression levels in vitro (Fig. 2c), highlighting a crucial role of the master regulators for the maintenance of MA-TAM-encoding gene expressions. As previous studies have explored the potential association between the density of GSCs with recruitment of TAMs [31, 32], we investigated whether MA-TAM-associated molecules could potentially drive mesenchymal transformation and the malignant cellular state of GSCs. As expected, treatment with either recombinant MARCO or CCL7 proteins significantly increased the expression levels of both mesenchymal- and stemness-associated markers, including CD44, homeobox protein NANOG (NANOG), leukemia inhibitory factor (LIF), and matrix metallopeptidase 2 (MMP-2) [33, 34] (Fig. 2d and Additional file 1: Figure S5A). Furthermore, both MARCO and CCL7 promoted the upregulation of tafazzin (TAZ), which has been suggested as a key transcriptional coactivator regulating mesenchymal trans-differentiation in malignant gliomas [7] (Fig. 2e and Additional file 1: Figure S5B).

Various cytokines released from TAMs modulate the cellular state and functional roles of adjacent GSCs and differentiated tumor cells in GBMs [13]. To interrogate the cellular effects of TAM-derived MARCO in vitro, we isolated TAMs from GBM PDX models based on the expression of MARCO and control monocytes from healthy mice. Afterwards, we generated conditioned media (CM) from monocyte, MARCO^low^, or MARCO^high^ TAMs and studied their effects on tumor cellular phenotypes. CM from MARCO^high^ TAMs significantly upregulated both invasive and angiogenic activities of GSCs and human umbilical vein endothelial cells (HUVECs), respectively (Fig. 2f, g) coupled with therapeutic resistance to irradiation (Fig. 2h, Additional file 1: Figure S6), which are representative characteristics of mesenchymal GBMs. Consistently, we discovered that MARCO^high^ TAM-derived CMs significantly elevated the transcriptional expression levels of mesenchymal-, stemness-, and invasion-associated molecules (Fig. 2l). Conversely, treatment of anti-MARCO antibodies significantly reduced the expression level of MARCO and prevented mesenchymal trans-differentiation of GSCs. (Additional file 1: Figure S7). Collectively, our results demonstrate that MARCO^high^ TAMs drive mesenchymal transformation and the aggressive phenotypes of GBM.

### MARCO^high^ TAMs accelerate tumor growth and promote mesenchymal phenotypes in vivo

**Fig. 3:**
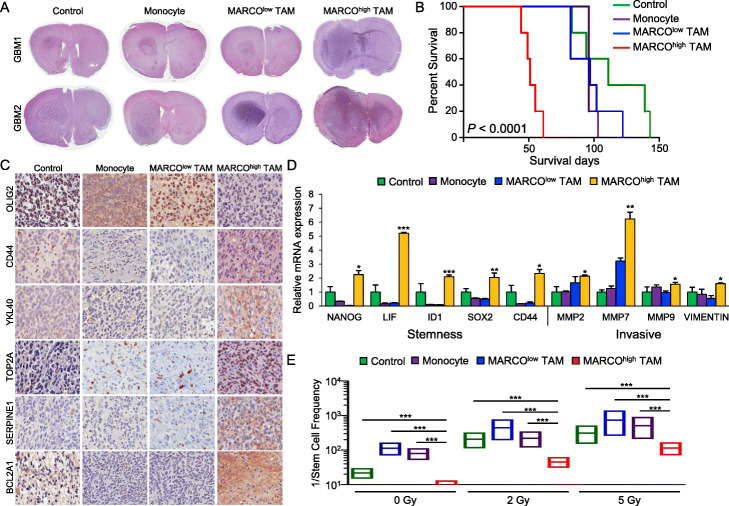
Effects of MARCO^high^ TAMs in vivo and ex vivo. **a** Representative hematoxylin and eosin staining of mouse brains orthotopically co-implanted with GSCs and either control, monocytes, MARCO^low^ TAMs, or MARCO^high^ TAMs. Each group was injected with 5 mice. **b** Kaplan-Meier survival curve analysis of the mouse models. **c** Representative immunohistochemical images of mesenchymal markers including CD44, YKL40, TOP2A, SERPINE1, and BCL2A1 in mouse models. **d** qRT-PCR analysis of stemness and invasive markers from ex vivo tumor specimens isolated from mouse models. **e** The 1/stem cell frequency of GSCs isolated from model mice and subjected to 2 or 5 Gy ionizing radiation. *P ≤ 0.05, **P ≤ 0.01, ***, *P* ≤ 0.001. Data shown in **c** and **e** are representative of two independent and reproducible experiments. Data shown in **d** are representative of three independent and reproducible experiments

TAMs and tumor cells generate a reciprocal feedback loop that leads to an immuno-suppressive and pro-tumorigenic environment. To evaluate whether MA-TAMs promote aggressive tumor growth in vivo, we generated orthotopic GBM PDX mouse models via co-injection of non-mesenchymal GSCs with either normal mouse monocytes, MARCO^low^ TAMs, or MARCO^high^ TAMs. Following intracranial implantation, we observed accelerated tumor engraftments followed by shortened survival span in PDX models that were co-injected with MARCO^high^ TAMs compared to either monocyte- or MARCO^low^-TAM-derived mouse models (Fig. 3a, b, Additional file 1: Figure S8A). Furthermore, immunohistochemical analyses demonstrated enrichments of key mesenchymal molecules including CD44, chitinase-3-like protein 1 (YKL-40), and topoisomerase II alpha (TOP2A) in non-mesenchymal GSCs that were co-injected with MARCO^high^ TAMs (Fig. 3c, Additional file 1: Figure S8B), which simultaneously promoted both invasive and stem-like properties of GSCs (Fig. 3d). Notably, ex vivo tumors originating from MARCO^high^-TAM PDX models demonstrated acquired resistance to irradiation, as shown by their enhanced sphere-forming capacity, suggesting that active crosstalk between GSCs and MARCO^high^ TAMs provides an innate ability for tumor cells to circumvent therapeutic vulnerability (Fig. 3e).

### Genomic correlates and cellular origins of MA-TAMs

**Fig. 4:**
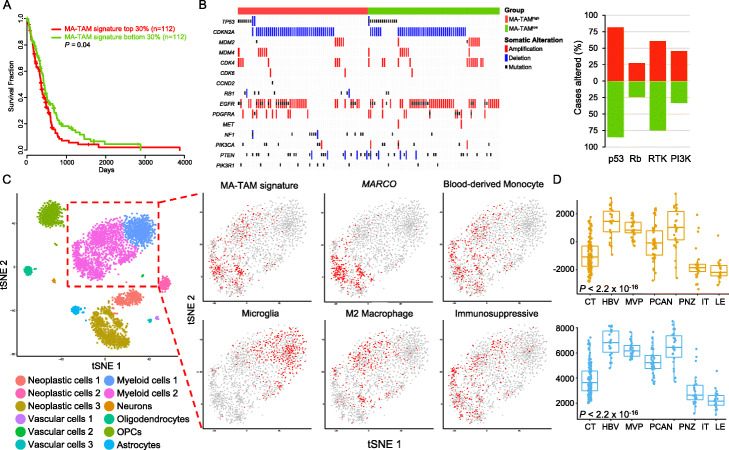
Genomic correlates of MA-TAM and its association with cellular origination and polarization state. **a** Kaplan-Meier survival curve analysis of 373 GBM patients based on MA-TAM signature score. **b** Genomic landscape of 133 MA-TAM^high^ and MA-TAM^low^ tumors (left panel). Somatic mutations, including single-nucleotide variations (SNVs), and small insertions/deletions, and copy number alterations, are shown. All somatic mutations with an allele frequency of > 5% are shown. Percentage of altered cases in key GBM driver pathways (right panel). **c** tSNE analysis of 3589 single-cell transcriptomes. Cell clusters are differentially colored and annotated based on each distinct cellular compartment (left panel). Expression of cell-type-specific signatures, polarization state, microenvironmental state overlaid on the tSNE space (right panel). **d** MA-TAM signature scores (upper panel) and master regulator activity (lower panel) in IVY Glioblastoma Atlas Project (IGAP) dataset. Cellular Tumor (CN; *n* = 111), hyperplastic blood vessels (HBV; *n* = 22), microvascular proliferation (MVP, *n* = 28), pseudo-palisading cells around necrosis (PCAN, *n* = 40), peri-necrotic zone (PNZ, *n* = 26), infiltrating tumor (IT, *n* = 24), and leading edge (LE, *n* = 19). *P ≤ 0.05, **P ≤ 0.01, ***P ≤ 0.001

To evaluate whether MA-TAM encoding genes and their master regulators reflect overall clinical prognosis in GBM, we stratified primary GBM patients based on their MA-TAM signature scores from TCGA dataset [35]. Survival analysis revealed that the MA-TAM signature and its master regulator activity were significantly correlated with reduced clinical outcomes (Fig. 4a, Additional file 1: Figure S9). We then sought to identify genomic correlates, including mutations (single nucleotide variations and small insertions/deletions) and copy number alterations, that were significantly enriched in tumors with high MA-TAM infiltration. Consistent with previous reports, MA-TAM^high^ tumors exhibited enrichments of *NF1* genomic aberrations, which has been speculated to drive recruitment of TAMs and microglial cells [4]. Although statistically not significant, we also discovered that MA-TAM^high^ tumors showed more frequent dysregulation in the phosphoinositide 3-kinase (PI3K) pathway, marked by genomic alterations in the phosphatase and tensin homolog (*PTEN*), phosphatidylinositol-4,5-bisphosphate 3-kinase catalytic subunit alpha (*PIK3CA*), and phosphoinositide-3-kinase regulatory subunit 1 *(PIK3R1)* genes (45% vs. 32%; Fig. 4b). Previous studies have postulated that dysregulation of the PI3K pathway, specifically via *PTEN* loss, generates an immunosuppressive environment [36, 37]; thus, we suspect that infiltration by MA-TAMs could potentially contribute to such malignant state as well. Conversely, MA-TAM^low^ tumors primarily demonstrated activation of various receptor tyrosine kinase (RTK)-encoding molecules, including epidermal growth factor receptor (*EGFR*), platelet-derived growth factor receptor alpha (*PDGFRA*), and MET proto-oncogene (*MET*) (75% vs. 60%; Fig. 4b).

To dissect and delineate the transcriptional profiles of MA-TAMs at a single-cell resolution, we curated gene expression profiles of 3589 single cells, comprising both neoplastic and non-neoplastic cells, using publicly available datasets [38]. Using the previously established t-distributed stochastic neighbor embedding (tSNE) parameters, we confirmed twelve distinct cell type clusters and evaluated the transcriptional expression levels of MA-TAM signatures in the myeloid cell compartments (Fig. 4c). Notably, myeloid cells were segregated into two distinct clusters based on the expression of MA-TAM-associated genes. Of the two primary myeloid clusters, we discovered significant co-enrichment of the MA-TAM signature with *MARCO* and blood-derived monocytes, suggesting that MA-TAMs mainly originated from peripheral blood. MA-TAM-positive cells also demonstrated enrichment of M2 macrophage signature and immunosuppressive environment, further consolidating their pro-tumorigenic potential (Fig. 4c). Using another publicly available single-cell dataset, we have determined whether the expression level of the MA-TAM signature was inherent in mesenchymal tumors [39]. As suspected, PJ017 and PJ032 tumors demonstrated predominance of mesenchymal tumor cells with enrichments of MA-TAM signature activity (Additional file 1: Figure S10). Furthermore, we have performed scTHI (single cell Tumor-Host Interaction) to identify significantly activated ligand-receptor interactions between mesenchymal tumor cells and *MARCO*+ TAMs. Notably, we have detected a total of 598 interactive pairs, among which 20 of them were involved in secreted factor encoding molecules, including *CCR1-CCL5*, *CCR5-CCL5*, *SPP1-CD44*, *CSF1R-CSF3*, and *SPP1-ITGA5*. (Additional file 1: Figure S11). Our results were further consolidated by cytokine array-based characterization where conditioned media that are derived from MARCO^high^ TAMs demonstrated high expression levels of SPP1, CCL5, CCL12, CXCL10, CXCL16, G-CSF, and MPO (Additional file 1: Figure S12).

The anatomical structure of GBM includes unique cellular compartments in the tumor microenvironment, and the intra-tumor heterogeneity is accompanied by distinct gene expression patterns [40–42]. To further interrogate the cellular origin of MA-TAMs, we quantified the expression levels of the MA-TAM signature using the Ivy Glioblastoma Atlas Project (IGAP) dataset [42]. Interestingly, the expression of both MA-TAM encoding genes and their transcriptional master regulators were highly enriched in cell populations that were derived from peri-necrotic and vascular zones, which are known to be the primary locations of blood-derived monocytes (Fig. 4d, Additional file 1: Figure S13).

## Discussion

Current immunotherapeutic strategies primarily focus on exploitation of immune-checkpoint molecules in cytotoxic T cells to combat tumor progression [43]. While a subset of immune cells is customarily tumoricidal, others adopt a protumoral phenotype that is attributable to tumor malignant transformation. Given the recognition of diverse immune cell population in TME, identifying a key immunomodulatory in each disease entity has become the utmost importance. GBM is a unique disease that is often devoid of lymphoid cells, and TAMs constitute a major source of innate immune cells in the GBM environment and control key cellular dynamics leading to the evasion of immune surveillance functions [41, 44–46]. Recent studies have highlighted dynamic interactions between pro-tumorigenic TAMs and GSCs that lead to global immuno-suppressive environment driving GBM pathogenesis [41, 45]. For example, GSCs preferentially secrete various cytokines to recruit monocyte-derived TAMs, which subsequently promote GSC maintenance, therapeutic resistance, and tumor recurrence [22, 24, 41, 47]. Extensive research has outlined the prevalence of TAM infiltration, specifically in mesenchymal GBMs [4, 46, 48]; thus, the molecular characterization of MA-TAM populations could provide new therapeutic windows for improving tumor-specific immunity. In the present study, we identified global transcriptional regulatory networks in MA-TAMs that drive mesenchymal transformation and malignancy in GSCs. Using reverse-engineering algorithms, we discovered a set of master regulators that modulate the transcriptional activity in MA-TAM encoding molecules. We also demonstrated that the upregulation of MA-TAM-associated gene signatures was significantly correlated with mesenchymal trans-differentiation during GBM evolutionary dynamics and identified *MARCO* as one of the most robustly expressed genes in MA-TAMs.

MARCO is a class A scavenger receptor that has been involved in essential macrophage programs, including phagocytosis and inflammation. Recent reports have recognized MARCO as a potential therapeutic target that is often overexpressed and involved in tumor microenvironment composition and promote poor prognosis across multiple cancer types. Furthermore, the expression level of MARCO could be upregulated through activation of TGF-β and IL-10, which subsequently generates immunosuppressive environment. We discovered that treatment with MARCO^high^ TAM-derived CM transformed non-mesenchymal GSCs into a more aggressive cellular state, manifesting key mesenchymal characteristics including increased cellular invasion, migration, and therapeutic resistance to ionizing radiation. The co-implantation of MARCO^high^ TAMs with non-mesenchymal GSCs led to transcriptome profiles similar to those of mesenchymal tumor cells, modulating the essential invasive and stem cell-like properties of GSCs. Furthermore, MARCO^high^ TAMs demonstrated significantly accelerated tumor growth and reduced survival rate in patient-derived xenograft models. Ex vivo tumor cells also developed global resistance to irradiation, as shown by their enhanced clonogenic growth. Moreover, we discovered that both MA-TAM transcriptional regulators and their target genes were associated with significantly worse prognosis in GBM patients. Furthermore, MA-TAM^high^ tumors were marked by frequent genomic ablations of *NF1* and PI3K pathway components, indicating the essential role of pathogenic activation of downstream effectors mediated by loss of NF1 and PI3K/AKT/mTOR signaling in GBM tumor cells for the induction and maintenance of MA-TAMs in GMB tumor microenvironment [4, 46, 48–50]. Single-cell analysis suggested the potential cellular origin of MA-TAMs as blood-derived monocytes, as well as co-enrichments of MA-TAMs with M2 macrophages and immunosuppressive environment. Notably, MARCO, a key protein within a subset of macrophages that are involved in phagocytosis, inflammation, and others [51], has been implicated as a new immune target for anti-TAM treatment for treating various solid tumors, including non-small-cell lung carcinoma, melanoma, and breast cancer [52].

In conclusion, our findings provide novel insights into the dynamic regulatory networks of MA-TAMs and their profound effects in GBM pathogenesis. As the infiltration of pro-tumorigenic TAMs is a key hallmark of tumor propagation, progression, and response to therapy, in-depth molecular studies of MA-TAM encoding molecules and their master regulators warrant further investigation to facilitate future immunotherapeutic approaches to treat GBM.

## Materials and methods

### Glioblastoma patient-derived specimens and primary cell culture

GBM tumor specimens and corresponding clinical records were obtained from patients undergoing surgery at the Samsung Medical Center (Seoul, Korea) in accordance with the guidelines of the institutional review board. Informed consent was obtained from each patient prior to the study. This work was performed in compliance with all relevant ethical regulations for research using human specimens. Surgical samples were either snap-frozen using liquid nitrogen for genomic analysis or enzymatically dissociated into single cells using Liberase TM (Roche Diagnostics, Basel, Switzerland) and cultured in neurobasal medium (Gibco, Waltham, MA, USA).

### Isolation of tumor-associated macrophages

As reported previously [11, 23–25], fresh tumor tissue specimens were subjected to FACS to isolate CD45+/CD11b+ TAMs. CellQuest Acquisition (BD Pharmingen, San Jose, CA, USA) and FlowJo software (Tree Star, Ashland, OR, USA) were used to acquire and quantify the intensity and distribution of fluorescence signals.

### Whole-transcriptome sequencing

RNA-seq libraries were prepared using the Illumina TruSeq RNA Sample Prep kit. Sequenced reads were mapped onto the reference human genome (hg19) using GSNAP. The initial BAM alignment files were sorted and summarized into BED files using SAMtools and bedTools. The BED files were used to calculate the reads per kilobase of transcript per million reads (RPKM) values for each gene using the DEGseq package.

### MA-TAM gene expression signature

Gene expression profiles of nine human TAM samples were subjected to gene signature extraction. Genes with maximum log2(RPKM+ 1) values < 1 were removed, and the resulting 12,497 genes were evaluated to identify positive correlations with GBM-intrinsic mesenchymal activity in the corresponding tumor cells. A total of 614 genes (*P* < 0.05 and *r* > 0.7) were further subjected to identify bona fide tumor-associated stromal/immune genes based on transcriptome analysis of orthotopic GBM PDX models. A total of 105 genes were selected to represent the MA-TAM-specific gene signature.

### MA-TAM master regulator analysis

To identify master regulators of the MA-TAM gene signature, gene expression profiles were curated from MA-TAMs and non-MA-TAMs and assembled into a transcriptional network using the RGBM algorithm developed in our previous works [28, 29, 53]. RGBM algorithm integrates the active binding network and gene expression profiles using a machine learning framework based on gradient boosting machines as detailed in [29]. Gene Set Enrichment Analysis (GSEA) was used to identify network hubs whose regulon was significantly enriched with the MA-TAM siganture. First, an active binding network was constructed and used as an a priori mechanistic network of connections between transcription factors (TFs) and their target genes. The active binding network was reconstructed for 2532 unique motif position weight matrices corresponding to 1203 unique TFs to identify 6,897,782 binding sites in the promoter regions of 12,985 targets (± 5 kb from the transcription start site) using the “Find Individual Motif Occurrences” tool (*P* < 1 × 10^−5^, average width 13.5 bp, hg19 assembly). These binding sites were overlapped with the open/active chromatin state (TssA, TssFlnkm, TssFlnkU, TssFlnkD, Tx, EnhG1, EnhG2, EnhA1m, and EnhA1) as determined using ChromHMM v.1.10 across 98 epigenomes from the Roadmap Epigenomics project (18-state model). The number of non-overlapping sites that overlapped with at least 1 bp of the previously identified 6,897,782 motif sites was 399,187 (hg19; average width 2840 bp), which corresponded to 1,874,570 unique TF-target (Motif + Enhancer) connections between 434 TFs and 12,985 targets. The reduced number of TFs was due to gene symbol consolidation among different data sources. The active binding network consisted of 6,652,518 overlapping active sites corresponding to 1,959,125 unique TF associations between 1779 TFs and 51,705 targets.

### Orthotopic GBM PDX mouse models

All mouse experiments were performed according to the guidelines of the Animal Use and Care Committees at Samsung Medical Center and the Association for Assessment and Accreditation of Laboratory Animal Care-accredited guidelines. Female 6–8-week-old BALB/c nude mice were used for intracranial implantation. Patient-derived glioma stem cells (1 × 10^5^ per mouse) and monocytes or TAMs (1 × 10^5^ per mouse) were co-injected into the brains of mice by stereotactic intracranial injection (coordinates: 2 mm anterior, 2 mm lateral, 2.5 mm depth from the dura). Mice were sacrificed either when they showed 25% body weight loss or when neurological symptoms (lethargy, ataxia, and seizures) were observed.

### Immunohistochemistry

Orthotopic PDXs were fixed with 4% paraformaldehyde in phosphate-buffered saline (PBS; Gibco) and embedded in paraffin. Sectioned slides were blocked with 10% horse serum and permeabilized with 0.3% Triton X-100 (Sigma-Aldrich, St. Louis, MO, USA). Samples were probed with primary antibodies against the following proteins: CD68 and YKL-40 (Abcam, Cambridge, UK), CD163 (Santa Cruz Biotechnology, Dallas, TX, USA), oligodendrocyte TF 2 (R&D Systems, Minneapolis, MN, USA), CD44 (Sigma-Aldrich), B cell lymphoma 2-related protein A1 and Top2A (LSBio, Seattle, WA, USA), and serpin family E member 1 (Novus Biologicals, Centennial, CO, USA). Immunoreactivity was quantified using a Tissue FAXS system (Tissuegnostics USA, Tarzana, CA, USA). Scanned images were analyzed with HistoQuest cytometry software. The threshold signal intensity was determined relative to the negative control.

### Real-time reverse-transcriptase PCR

Total RNA was isolated using RNeasy Mini kit and cDNA was synthesized using RT2 First Strand kit (Qiagen). Quantitative reverse-transcriptase PCR (qRT-PCR) was performed on the 7900HT Fast Real-time PCR system (Applied Biosystems, Foster City, CA, USA) using RT2 SYBR Green qPCR Mastermix (Qiagen, Hilden, Germany). The following primers were used: Human LIF forward (Fw) 5′-TCTTGGCGGCAGGAGTTG-3′, reverse (Rev) 5′-CCGCCCCATGTTTCCA-3′; Human NANOG Fw 5′-TTTGTGGGCCTGAAGAAAACT-3′, Rev. 5′-AGGGCTGTCCTGAATAAGCAG-3′; Human ID1 Fw 5′-CTACGACATGAACGGCTGTTACTC-3′, Rev. 5′-TGGCTCGGCCAGGACTAC-3′; Human SOX2 Fw 5′-TGCGAGCGCTGCACAT-3′, Rev. 5′-TCATGAGCGTCTTGGTTTTCC-3′; Human OLIG2 Fw 5′-ATAGATCGACGCGACACCAG-3′, Rev. 5′-ACCCGAAAATCTGGATGCGA-3′; Human SERPINE1 Fw 5′-ACCGCAACGTGGTTTTCTCA-3′, Rev. 5′-TTGAATCCCATAGCTGCTTGAAT-3′; Human CD44 Fw 5′-AGAAGGTGTGGGCAGAAGAA-3′, Rev. 5′-AAATGCACCATTTCCTGAGA-3′; Human MMP8 Fw 5′-TCTTCCTCCACACACAGCTTG-3′, Rev. 5′-CTGCAACCATCGTGGCATTC-3′; Human β-actin Fw 5′-AGAAAATCTGGCACCACACC-3′, Rev. 5′-AGAGGCGTACAGGGATAGCA-3′; Mouse MARCO Fw 5′-GGGTCAAAAAGGCGAATCTTTC-3′, Rev. 5′-CCCTCTGGAGTAACCGAGCA-3′; Mouse CD180 Fw 5′-TAGGTCTCAATGAAATTCCTGGC-3′, Rev. 5′-AATCTGGCACCTGGTTAAATCC-3′; Mouse GPR18 Fw 5′-CACCCTGAGCAATCACAACCA-3′, Rev. 5′-AGTGACATTAACAAACAGCCCA-3′; Mouse KYNU Fw 5′-GTCAAGCCTGCGTTAGTGG-3′, Rev. 5′-CTCGCGGCAAGTCTTCAGAG-3′; Mouse TGM2 Fw 5′-GACAATGTGGAGGAGGGATCT-3′, Rev. 5′-CTCTAGGCTGAGACGGTACAG-3′; Mouse SPI1 Fw 5′-ACAGCATCTGGTGGGTGGAC-3′, Rev. 5′-GCCTGTCTTGCCGTAGTTGC-3′; Mouse PPARG Fw 5′-GCTGAACGTGAAGCCCATCG-3′, Rev. 5′-GGCGAACAGCTGAGAGGACT-3′; Mouse BATF Fw 5′-CAGCTTCAGCCGCTCTCCTC-3′, Rev. 5′-AGGGTGTCGGCTTTCTGTGT-3′; Mouse CCL7 Fw 5′-GGTGTCCCTGGGAAGCTGTT-3′, Rev. 5′-GCCTCCTCGACCCACTTCTG-3′; Mouse CD180 Fw 5′-GGCCTCCAATCGCATCAGCA-3′, Rev. 5′-GGCCTCCAATCGCATCAGCA-3′; Mouse MMP8 Fw 5′-GCCTCGATGTGGAGTGCCTG-3′, Rev. 5′-GGTGAAGGTCAGGGGCGATG-3′; Mouse CR2 Fw 5′-CTGCTTCGTGCCCTTCCACA-3′, Rev. 5′-CGCTGATGACTCGAGCCTGG-3′; Mouse GPR18 Fw 5′-ACCTGGAGTCAACCTCCCCC-3′, Rev. 5′-CATCCTGGCACTGGCTCTGG-3′; Mouse KYNU Fw 5′-AGGAGACTCGATCGCCGTGA-3′, Rev. 5′-ACAAAGGCACCAGCCAGACC-3′; Mouse S100A8 Fw 5′-GGAGAAGGCCTTGAGCAACC-3′, Rev. 5′-TGTGAGATGCCACACCCACT-3′; Mouse FPR3 Fw 5′-GCTGCCGAATCTGGGGAGTC-3′, Rev. 5′-TGGGAACAGCCTCTGGACGA-3′; Mouse PHACTR2 Fw 5′-TCTCGTCCCAGAGCACCCAA-3′, Rev. 5′-TTGCTTGTGGCCTGCTCCTC-3′; Mouse AREG Fw 5′-GAGAACTCCGCTGCTACCGC-3′, Rev. 5′-GAGAACTCCGCTGCTACCGC-3′; Mouse β-actin Fw 5′-ATGGTGGGAATGGGTCAGAA-3′, Rev. 5′-CCATGTCGTCCCAGTTGGTAA-3′.

### Generation of CM

A total of 1 × 10^6^ monocytes, MARCO^low^ TAMs, or MARCO^high^ TAMs were cultured in neurobasal medium for 18 h. The supernatants were harvested, centrifuged, and filtered through a 0.40-μm filter to obtain CM.

### 3D in vitro invasion assay

A microfluidic device was designed to evaluate the invasive potential of GSCs. GSCs from each CM-treated group were prepared at a density of 1.5 × 10^6^ cells/ml. After inducing the attachment of cells to the side of the collagen scaffolds by gravity, the device was placed in a vertical position in a humidified CO_2_ incubator at 37 °C for 2 h. After cell attachment, the culture medium was refreshed every 24 h for 3 days.

### Evaluation of radiation resistance via neurosphere-forming limiting dilution assay

For ex vivo analysis of GSCs co-injected with monocytes, MARCO^low^ TAMs or MARCO^high^ TAMs, PDX-derived tumors from each group were freshly dissociated into single cells and seeded in 96-well plates at 1–100 cells/well. Cells were treated with 5 Gy ionizing radiation using the IBL 437C blood irradiator (CIS Bio International, Saclay, France). After 2 weeks, each well was examined for neurosphere formation. The LDA clonogenic index is calculated as the inverse of the x-intercept of the regression between the number of wells without spheres and the number of cells seeded. The frequency of stem-like clonogenic cells was determined using Extreme Limiting Dilution Analysis (http://bioinf.wehi.edu.au/software/elda/).

### In vitro tube formation assay

The tube formation assay was carried out using HUVECs to evaluate the angiogenic effects of TAMs. First, growth factor-free Matrigel (Corning Inc., Corning, NY, USA) was added to a 96-well plate and incubated at 37 °C for 30 min. HUVECs were cultured with each of the previously prepared CMs. Cells from each group (2 × 10^4^/well) were seeded in the Matrigel-pre-coated wells and incubated in a 5% CO_2_ incubator. The formation of tubular structures was assessed within 4 h by measuring the tube length under a microscope (*N* = 3 fields/well) and was quantitated using ImageJ software.

### Multiplex IHC

Multiplex staining was performed using an Opal 7 Immunology Discover kit (OP7DS1001KT, PerkinElmer, Waltham, MA, USA) according to the manufacturer’s protocol. Slides were deparaffinized in xylene and rehydrated in ethanol. Antigen retrieval was performed in AR9 buffer by microwave treatment. Antibodies against the following proteins were used: CD68, PPARG, BATF, SPI1, MARCO, and YKL-40. The primary antibodies were incubated for 30 min in a humidified chamber at room temperature, followed by detection using a mouse/rabbit SuperPicture Polymer Detection Kit. The primary antibodies were visualized using Opal Fluorophore Working Solution, after which the slides were placed in AR9 buffer and again subjected to microwave treatment. The slides were examined using VECTRA 3.0 Automated Quantitative Pathology Imaging System (PerkinElmer). InForm image analysis software (PerkinElmer) was used to analyze the spectra of all fluorophores.

### Isolation of mouse peritoneal macrophages

Peritoneal macrophages were obtained under sterile conditions 3 days after the intraperitoneal injection of 1–5% thioglycolate into the abdominal cavity of nude mice. The cells were harvested by washing the peritoneal cavity with cold PBS (Gibco). Cells were centrifuged and resuspended in Dulbecco’s modified Eagle’s medium (DMEM; Gibco) supplemented with 10% fetal bovine serum and 1% penicillin/streptomycin (Sigma-Aldrich). The cells were allowed to adhere for 4 h, washed to remove non-adherent cells, and cultured in DMEM supplemented with 1% penicillin/streptomycin.

### Overexpression lentivirus production and infection

Overexpression constructs were generated into lentivirus. Briefly, 3–4 × 10^6^ 293 T cells were seeded in 100-mm culture dishes for 24 h prior to co-transfection with 4.5 μg of lentivirus construct (pHRST-IRES-Pparg), 3 μg of psPAX2 (Paired box gene 2), and 1.5 μg of pMD2.G using 27 μg of Lipofectamine 2000. The media were changed after 6 h and the supernatant containing the lentivirus was harvested 48 h after the transfection. Viral particles were concentrated and purified using a Lenti-X-concentrator. Cells were infected with lentivirus in the presence of 6 μg of polybrene. eGFP was used as a fluorescent marker to distinguish successfully infected populations. Empty construct was used as control for all experiments.

### Statistical analysis

All data were expressed as the mean ± standard deviation (SD) or ± standard error of the mean (SEM) based on at least two or three independent experiments. Student’s two-tailed *t* test or analysis of variance (ANOVA) was used to determine the statistical significance between two groups or more of continuous variables. Fisher’s exact test was used to determine the statistical significance between two groups of categorical data. Log-rank tests were used for survival analyses. A value of *P* < 0.05 was considered statistically significant.

## Supplementary information

**Additional file 1: Figure S1.** A schematic illustration on identification of MA-TAM signature and master regulators. **Figure S2.** MA-TAM target gene pathway enrichment. **Figure S3.** Global regulatory network of MA-TAM. **Figure S4.** Multi-color immunohistochemical images of MA-TAM encoding molecules. **Figure S5.** Effects of MARCO and CCL7 on mesenchymal markers. **Figure S6.** Effects of TAM-derived CM on GSC stemness in response to irradiation. **Figure S7.** Effects of anti-MARCO therapeutic antibodies. **Figure S8. ** In vivo effects of MARCO^high^ TAMs in PDX models. **Figure S9.** Clinical correlation of MA-TAM master regulators. **Figure S10.** Single cell analysis of MA-TAM signature. **Figure S11.** Transcriptome analysis of scTHI at single-cell resolution. **Figure S12.** Cytokine array-based characterization of MARCO^high^ TAMs. **Figure S13.** Anatomical expression of MA-TAM signature.

**Additional file 2.** Review History.

## Data Availability

All sequenced data have been deposited in the European Genome-phenome Archive (EGA) under accession EGAD00001003324 [54].
