# Supplementary material for: Transcriptional regulatory networks of tumor-associated macrophages that drive malignancy in mesenchymal glioblastoma
Source: Genome Biol. 2020 Aug 26;21:216. doi: 10.1186/s13059-020-02140-x (PMC7448990; doi:10.1186/s13059-020-02140-x)
Supplement: Supplementary file 1 — Additional file 1: Figure S1. A schematic illustration on identification of MA-TAM signature and master regulators. Figure S2. MA-TAM target gene pathway enrichment. Figure S3. Global regulatory network of MA-TAM. Figure S4. Multi-color immunohistochemical images of MA-TAM encoding molecules. Figure S5. Effects of MARCO and CCL7 on mesenchymal markers. Figure S6. Effects of TAM-derived CM on GSC stemness in response to irradiation. Figure S7. Effects of anti-MARCO therapeutic antibodies. Figure S8. In vivo effects of MARCOhigh TAMs in PDX models. Figure S9. Clinical correlation of MA-TAM master regulators. Figure S10. Single cell analysis of MA-TAM signature. Figure S11. Transcriptome analysis of scTHI at single-cell resolution. Figure S12. Cytokine array-based characterization of MARCOhigh TAMs. Figure S13. Anatomical expression of MA-TAM signature. [file 13059_2020_2140_MOESM1_ESM.pptx]

## Slide 1
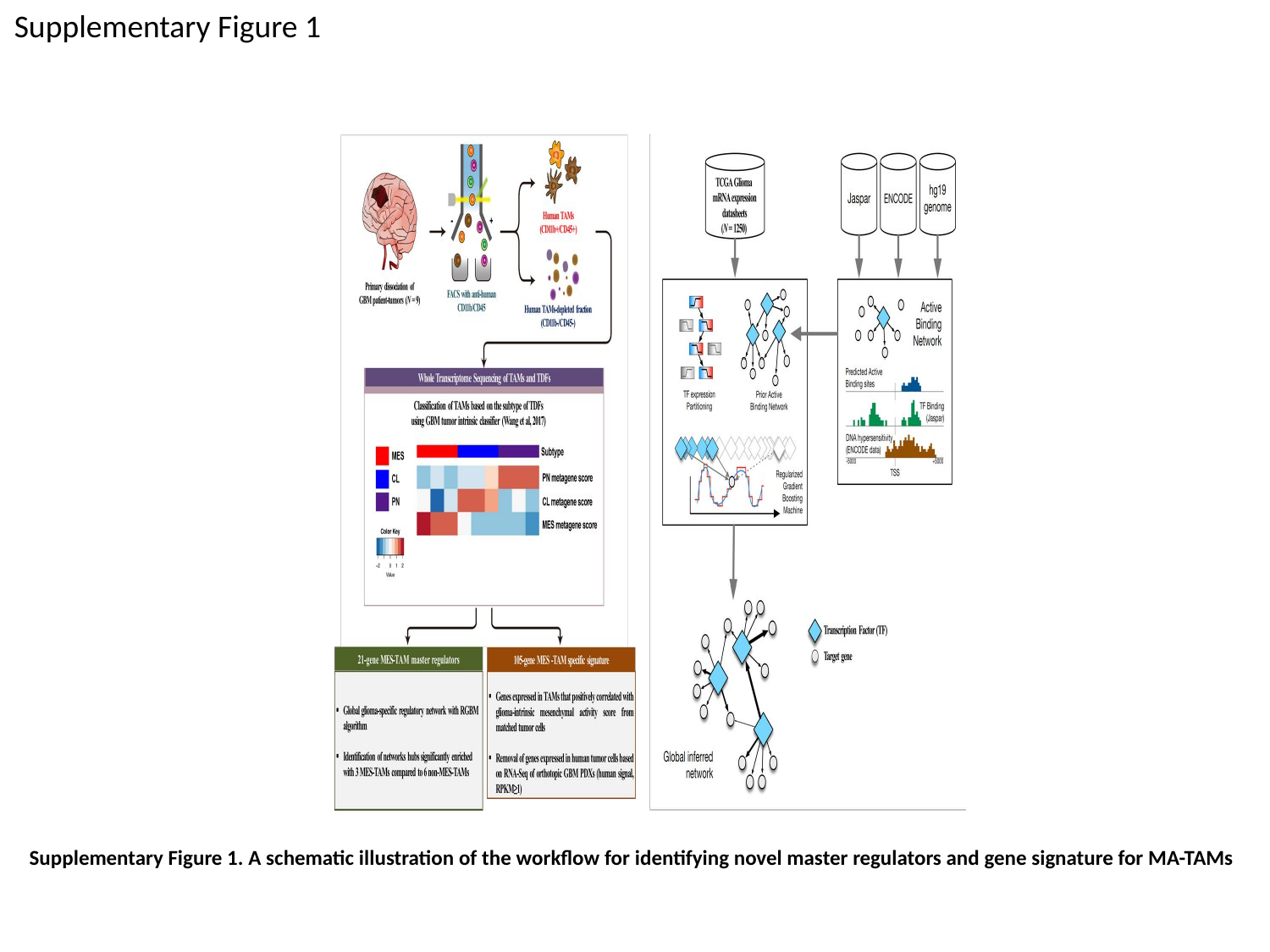

Supplementary Figure 1
Supplementary Figure 1. A schematic illustration of the workflow for identifying novel master regulators and gene signature for MA-TAMs

## Slide 2
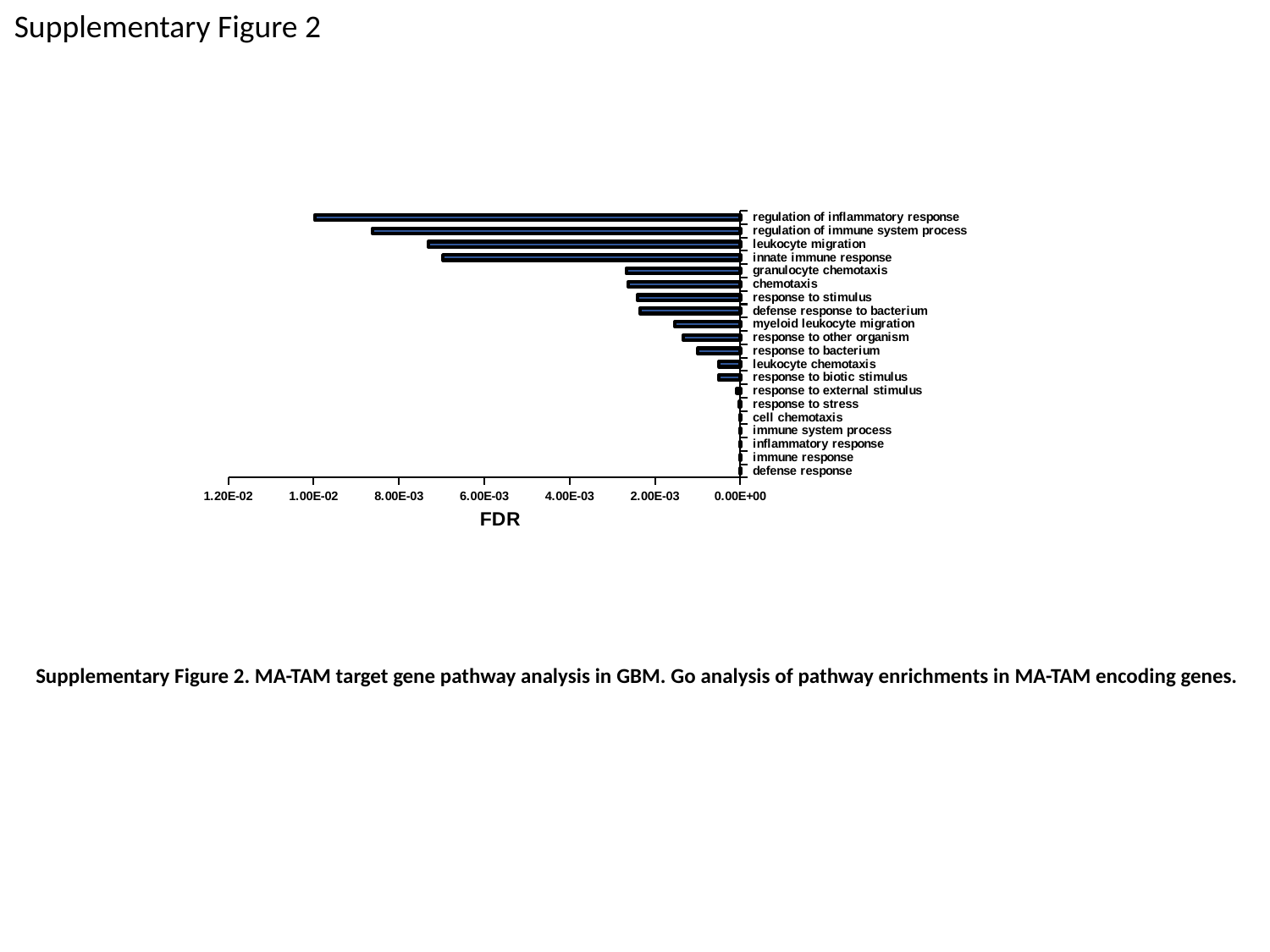

Supplementary Figure 2
### Chart
| Category | |
|---|---|
| defense response | 7.88e-10 |
| immune response | 9.68e-07 |
| inflammatory response | 2.28e-06 |
| immune system process | 4.87e-06 |
| cell chemotaxis | 8.47e-06 |
| response to stress | 2.74e-05 |
| response to external stimulus | 7.65e-05 |
| response to biotic stimulus | 0.000503 |
| leukocyte chemotaxis | 0.000503 |
| response to bacterium | 0.000996 |
| response to other organism | 0.00133 |
| myeloid leukocyte migration | 0.00153 |
| defense response to bacterium | 0.00235 |
| response to stimulus | 0.00241 |
| chemotaxis | 0.00262 |
| granulocyte chemotaxis | 0.00267 |
| innate immune response | 0.00697 |
| leukocyte migration | 0.00731 |
| regulation of immune system process | 0.00862 |
| regulation of inflammatory response | 0.00997 |Supplementary Figure 2. MA-TAM target gene pathway analysis in GBM. Go analysis of pathway enrichments in MA-TAM encoding genes.

## Slide 3
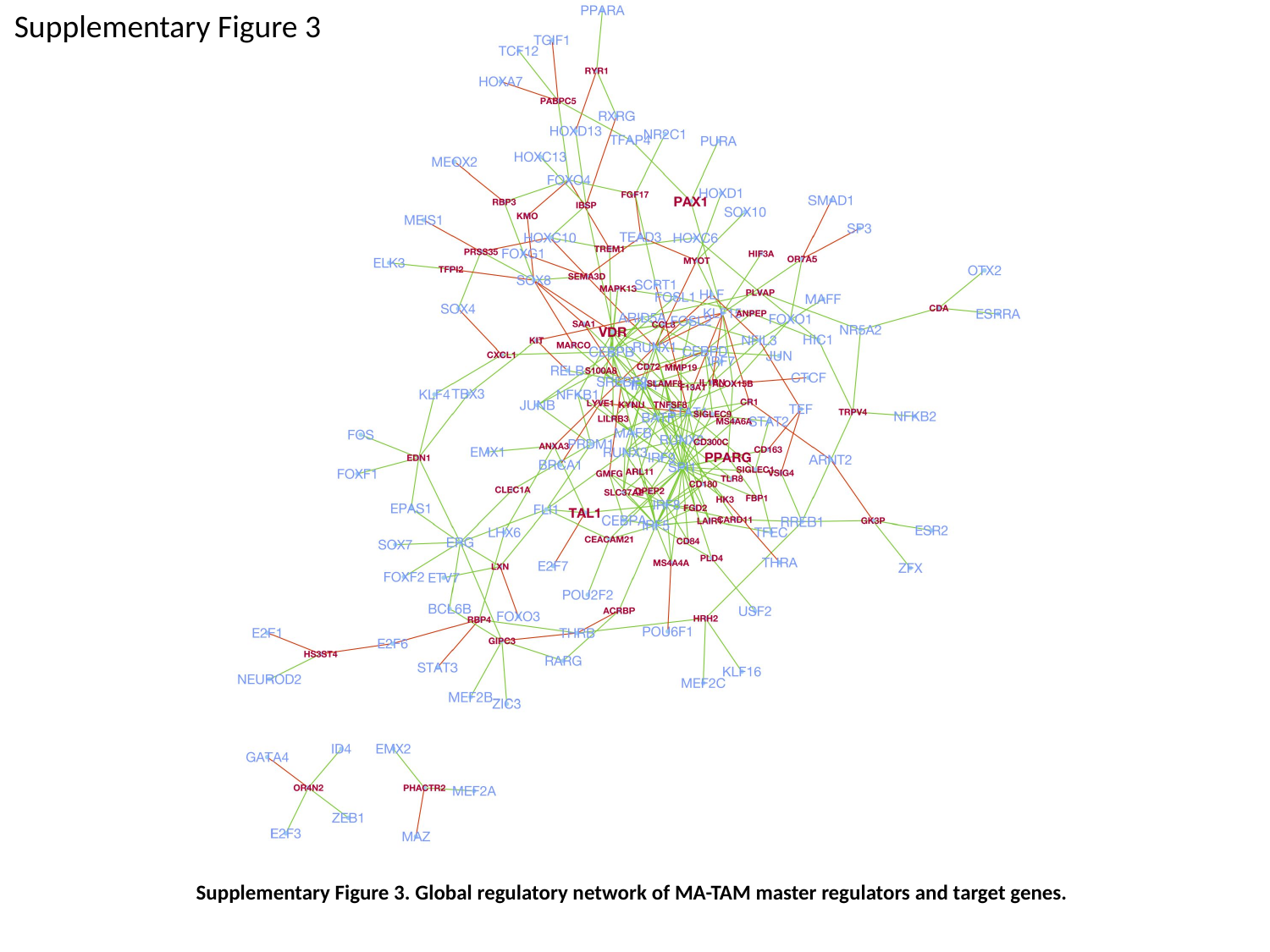

Supplementary Figure 3
Supplementary Figure 3. Global regulatory network of MA-TAM master regulators and target genes.

## Slide 4
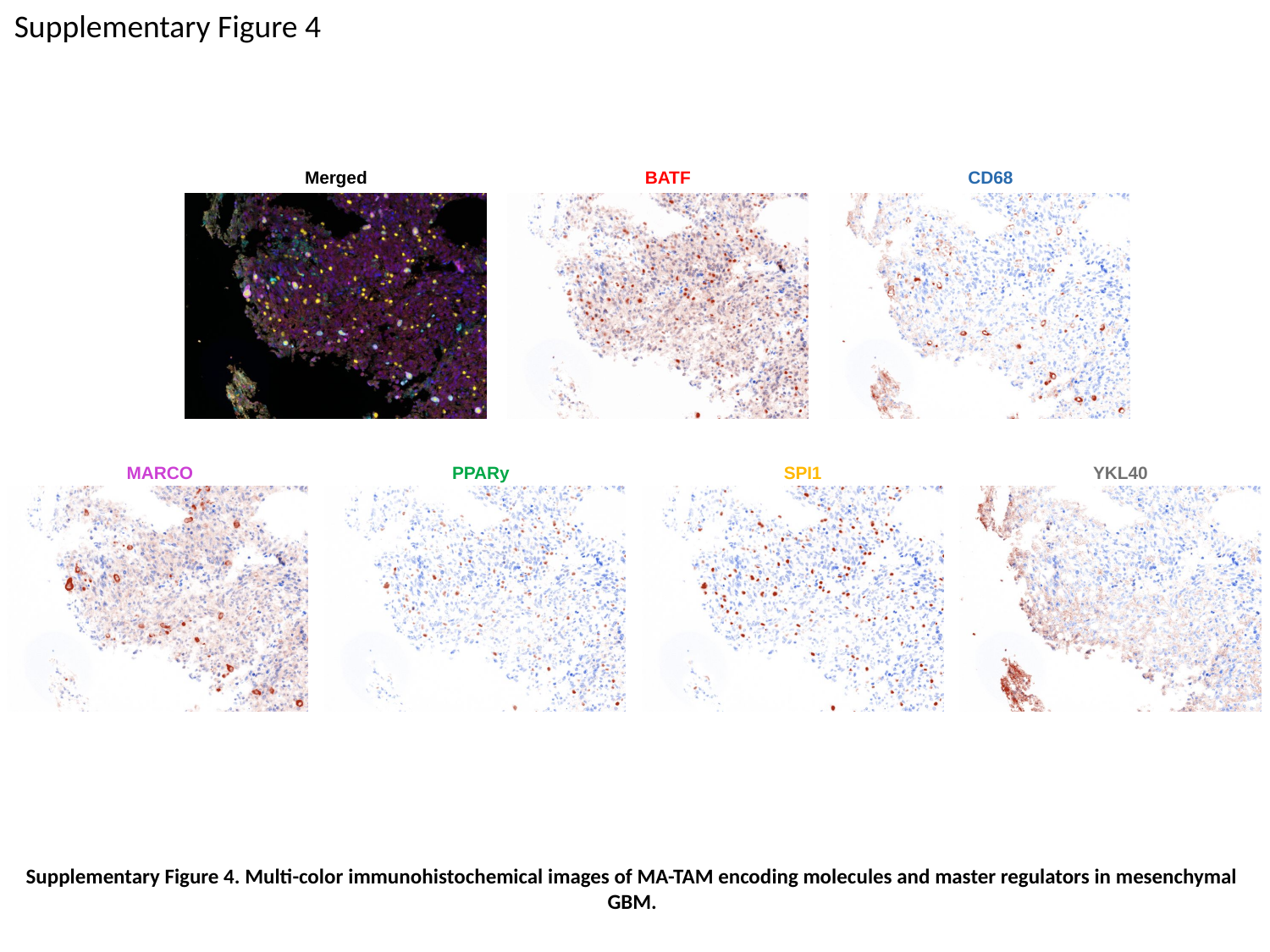

Supplementary Figure 4
Merged
BATF
CD68
MARCO
PPARy
SPI1
YKL40
Supplementary Figure 4. Multi-color immunohistochemical images of MA-TAM encoding molecules and master regulators in mesenchymal GBM.

## Slide 5
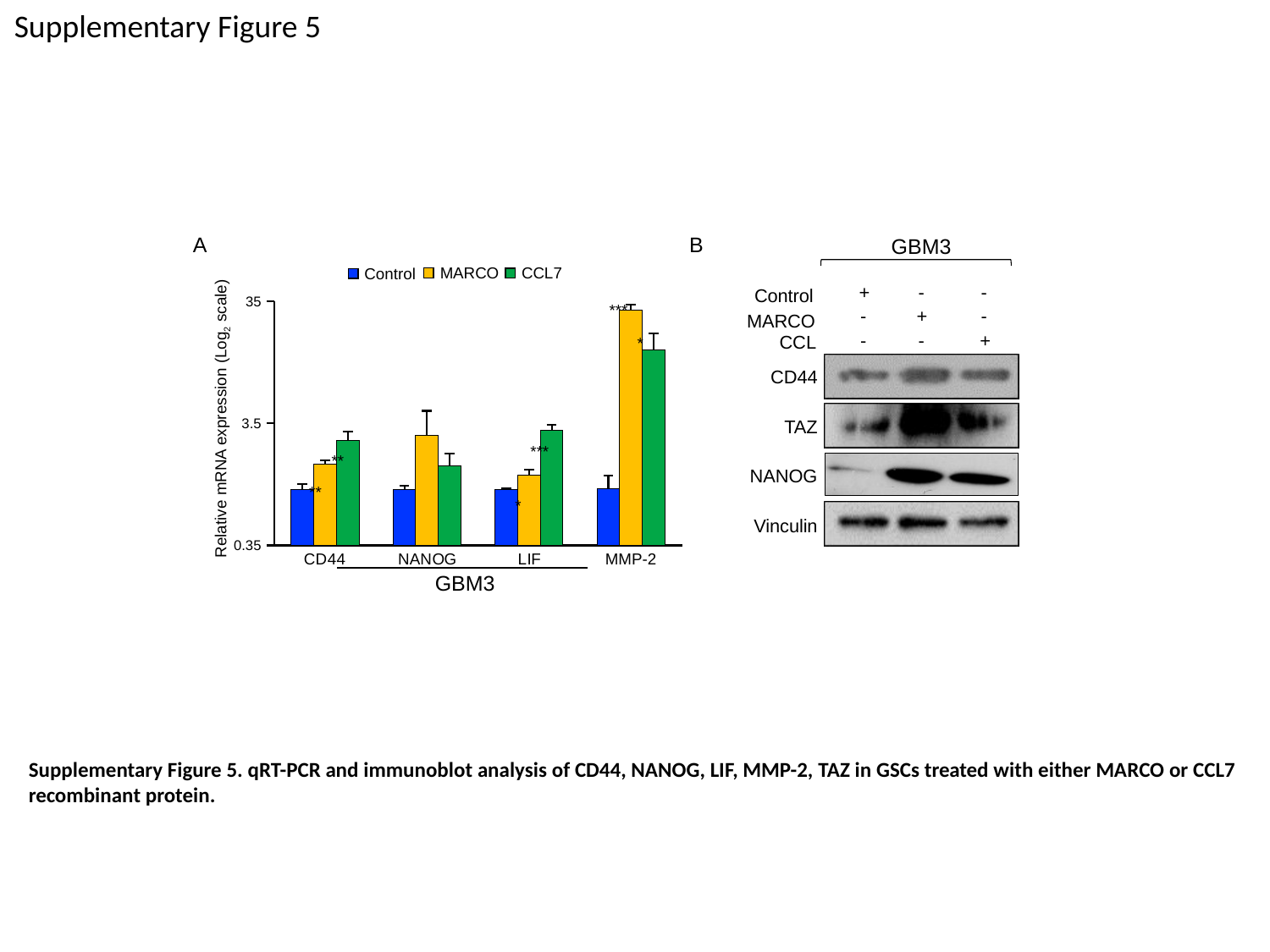

Supplementary Figure 5
B
A
GBM3
+
-
-
Control
-
+
-
MARCO
-
-
+
CCL
CD44
TAZ
NANOG
Vinculin
MARCO
CCL7
Control
### Chart
| Category | Control | MARCO | CCL7 |
|---|---|---|---|
| CD44 | 1.004284666666667 | 1.609428 | 2.534486 |
| NANOG | 1.000244466666667 | 2.768934 | 1.558438 |
| LIF | 1.000134 | 1.315537333333333 | 3.070685333333333 |
| MMP-2 | 1.023721666666667 | 29.69851666666667 | 13.907953 |***
*
Relative mRNA expression (Log2 scale)
***
**
**
*
GBM3
Supplementary Figure 5. qRT-PCR and immunoblot analysis of CD44, NANOG, LIF, MMP-2, TAZ in GSCs treated with either MARCO or CCL7 recombinant protein.

## Slide 6
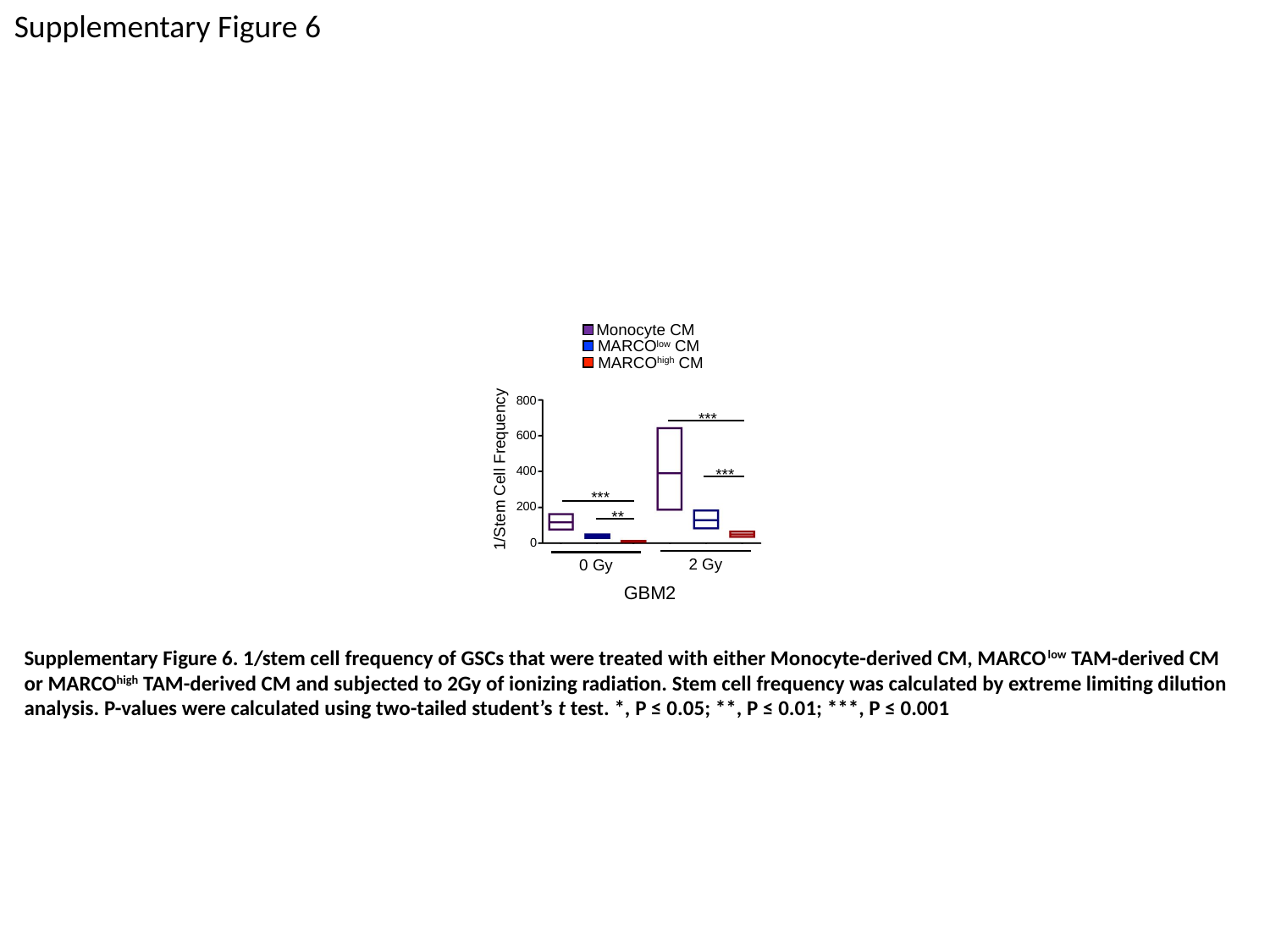

Supplementary Figure 6
Monocyte CM
MARCOlow CM
MARCOhigh CM
800
***
600
1/Stem Cell Frequency
400
***
***
200
**
0
2 Gy
0 Gy
GBM2
Supplementary Figure 6. 1/stem cell frequency of GSCs that were treated with either Monocyte-derived CM, MARCOlow TAM-derived CM or MARCOhigh TAM-derived CM and subjected to 2Gy of ionizing radiation. Stem cell frequency was calculated by extreme limiting dilution analysis. P-values were calculated using two-tailed student’s t test. *, P ≤ 0.05; **, P ≤ 0.01; ***, P ≤ 0.001

## Slide 7
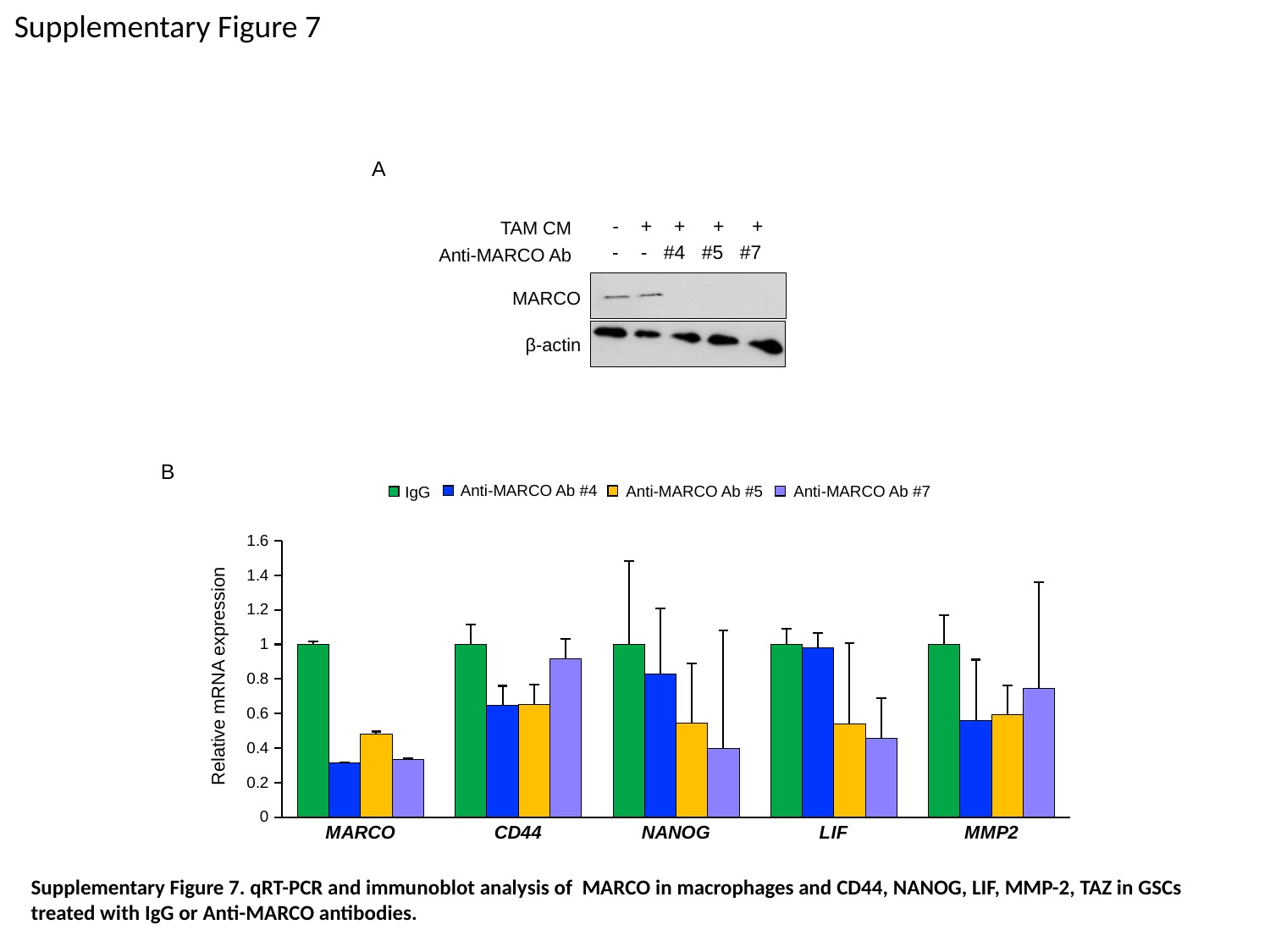

Supplementary Figure 7
A
 - + + + +
TAM CM
- - #4 #5 #7
Anti-MARCO Ab
MARCO
β-actin
B
Anti-MARCO Ab #4
Anti-MARCO Ab #5
Anti-MARCO Ab #7
IgG
### Chart
| Category | CM | anti-MARCO ab #4 | anti-MARCO ab #5 | anti-MARCO ab #7 |
|---|---|---|---|---|
| MARCO | 1.0 | 0.313285544560274 | 0.480492808403395 | 0.334234587958917 |
| CD44 | 1.0 | 0.645192684629082 | 0.65403386256681 | 0.917348381158478 |
| NANOG | 1.0 | 0.829397509398275 | 0.544206053584644 | 0.396825594430023 |
| LIF | 1.0 | 0.981989896272915 | 0.537509870313002 | 0.454258210776801 |
| MMP2 | 1.0 | 0.558920507640959 | 0.594326468909695 | 0.743478611965351 |Relative mRNA expression
Supplementary Figure 7. qRT-PCR and immunoblot analysis of MARCO in macrophages and CD44, NANOG, LIF, MMP-2, TAZ in GSCs treated with IgG or Anti-MARCO antibodies.

## Slide 8
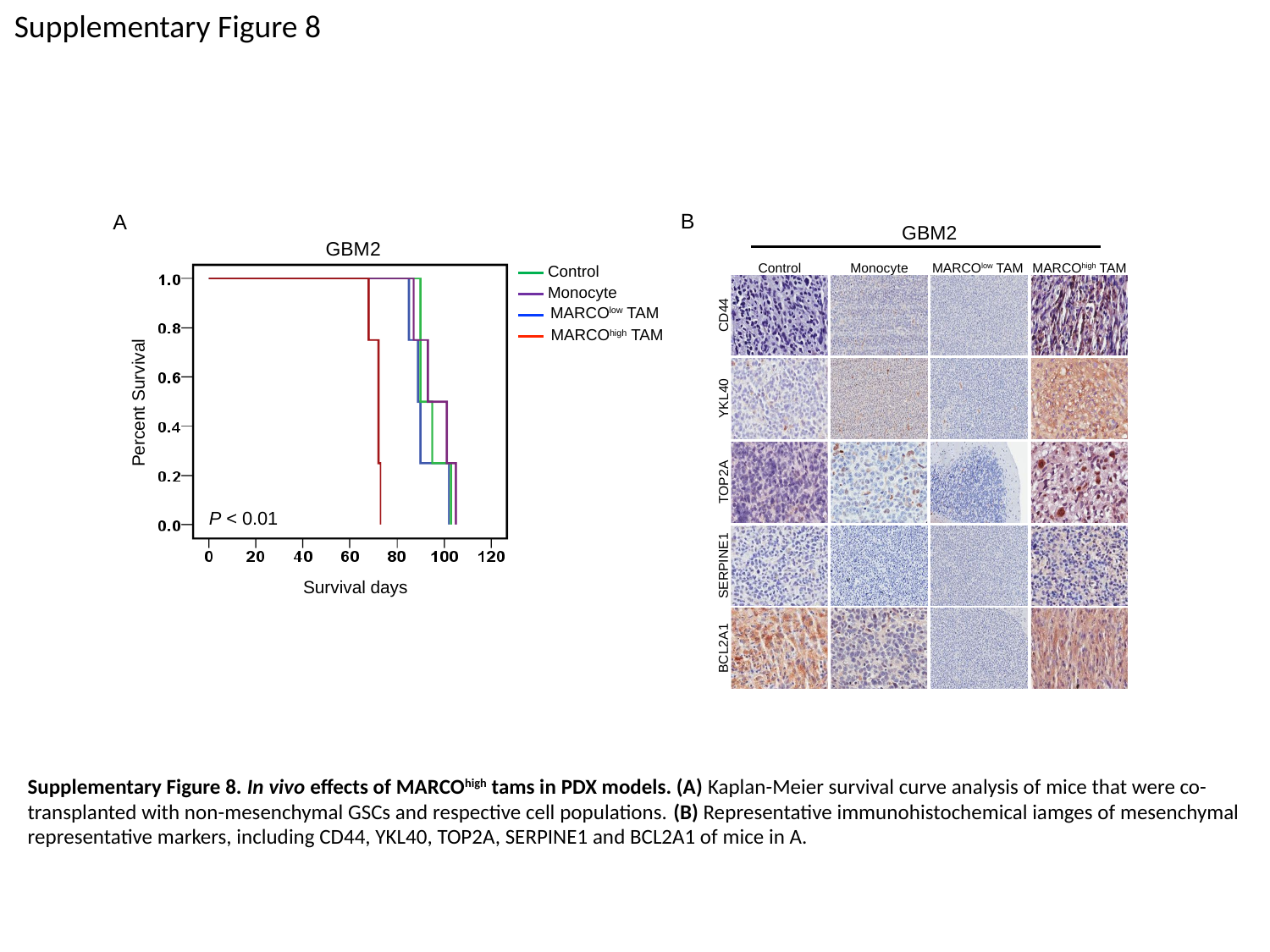

Supplementary Figure 8
B
A
GBM2
Control
Monocyte
MARCOlow TAM
MARCOhigh TAM
CD44
YKL40
TOP2A
SERPINE1
BCL2A1
GBM2
Control
Monocyte
MARCOlow TAM
MARCOhigh TAM
Percent Survival
P < 0.01
Survival days
Supplementary Figure 8. In vivo effects of MARCOhigh tams in PDX models. (A) Kaplan-Meier survival curve analysis of mice that were co-transplanted with non-mesenchymal GSCs and respective cell populations. (B) Representative immunohistochemical iamges of mesenchymal representative markers, including CD44, YKL40, TOP2A, SERPINE1 and BCL2A1 of mice in A.

## Slide 9
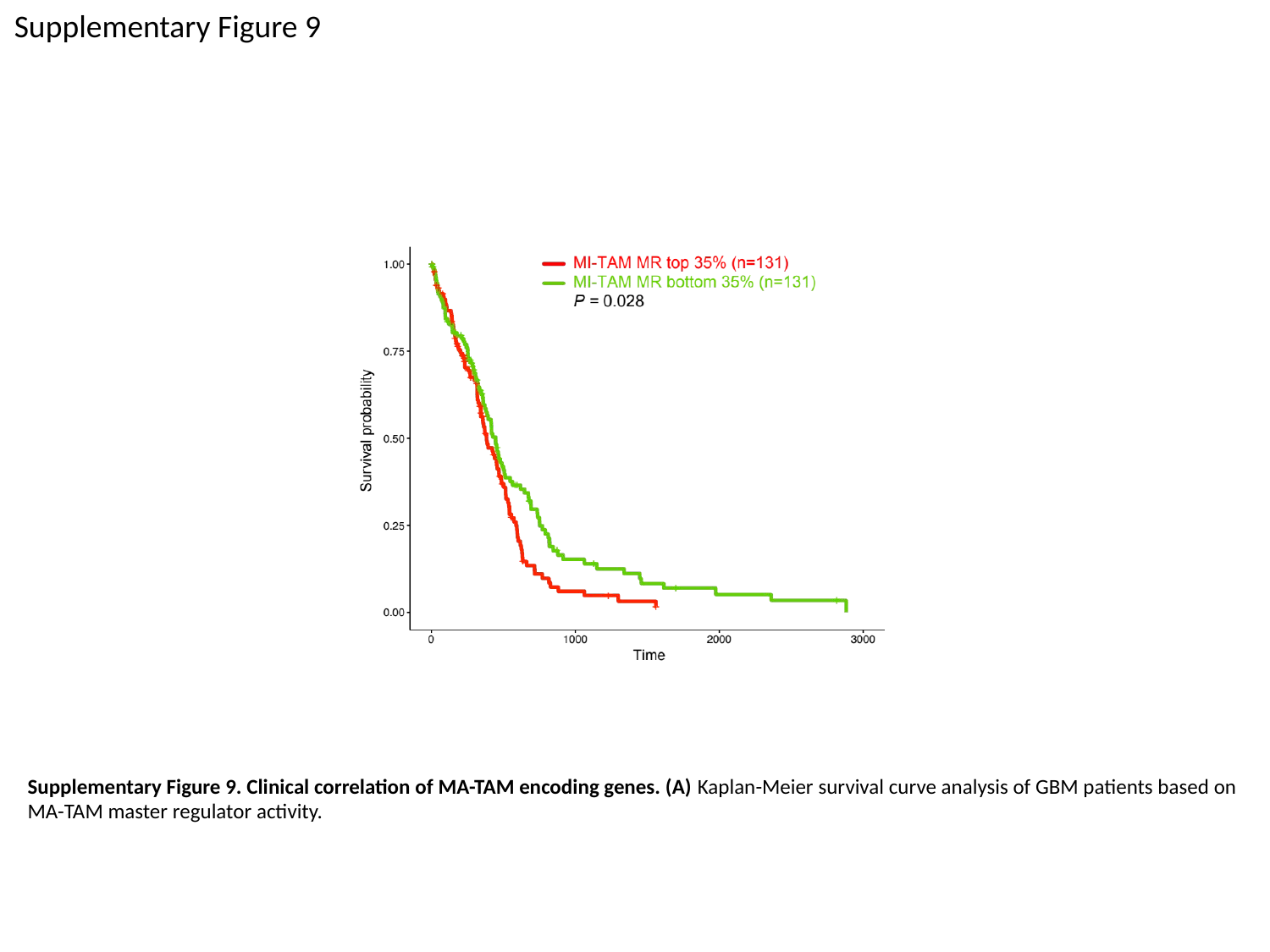

Supplementary Figure 9
Supplementary Figure 9. Clinical correlation of MA-TAM encoding genes. (A) Kaplan-Meier survival curve analysis of GBM patients based on MA-TAM master regulator activity.

## Slide 10
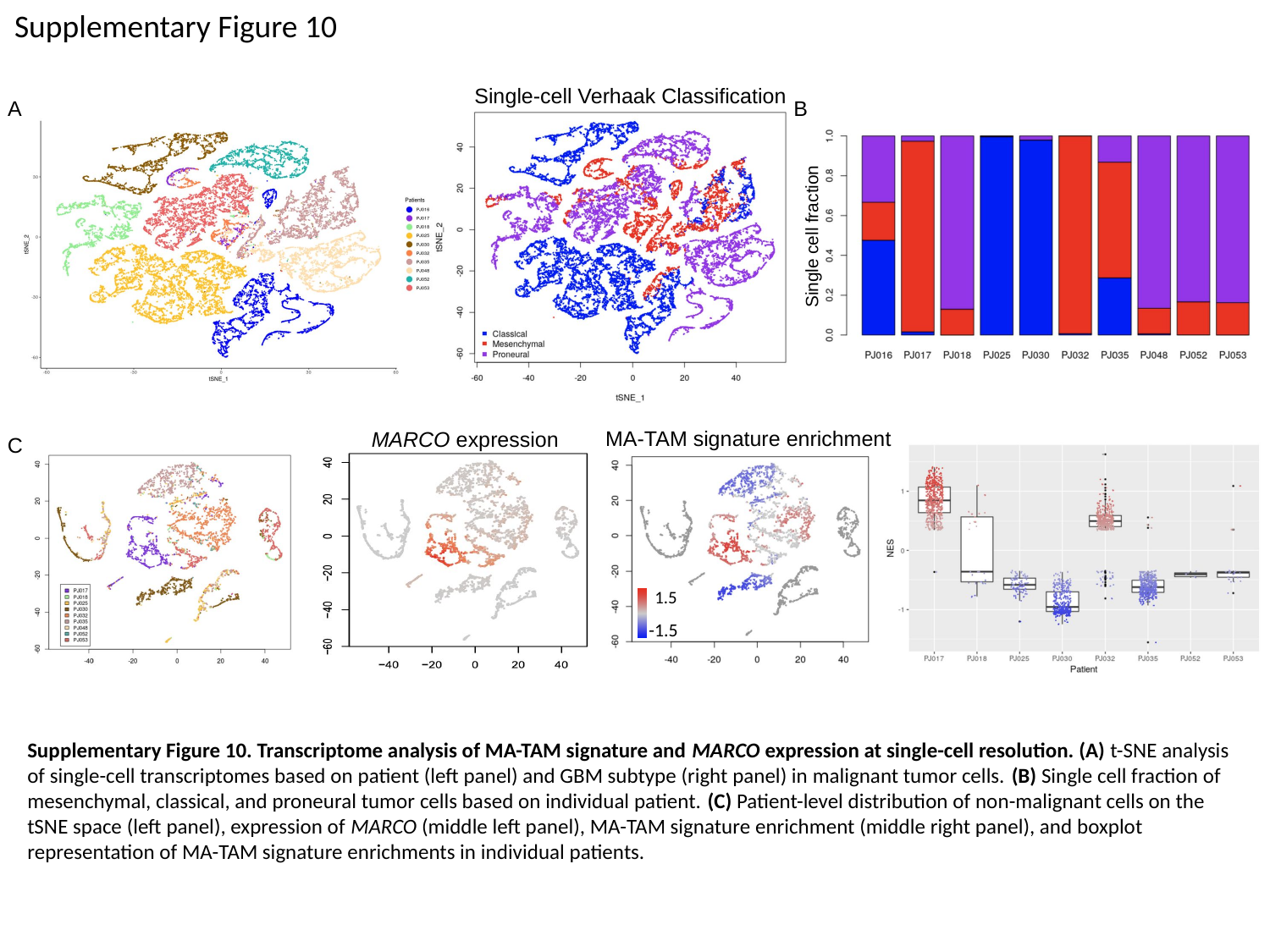

Supplementary Figure 10
Single-cell Verhaak Classification
B
A
Single cell fraction
MA-TAM signature enrichment
MARCO expression
C
1.5
-1.5
Supplementary Figure 10. Transcriptome analysis of MA-TAM signature and MARCO expression at single-cell resolution. (A) t-SNE analysis of single-cell transcriptomes based on patient (left panel) and GBM subtype (right panel) in malignant tumor cells. (B) Single cell fraction of mesenchymal, classical, and proneural tumor cells based on individual patient. (C) Patient-level distribution of non-malignant cells on the tSNE space (left panel), expression of MARCO (middle left panel), MA-TAM signature enrichment (middle right panel), and boxplot representation of MA-TAM signature enrichments in individual patients.

## Slide 11
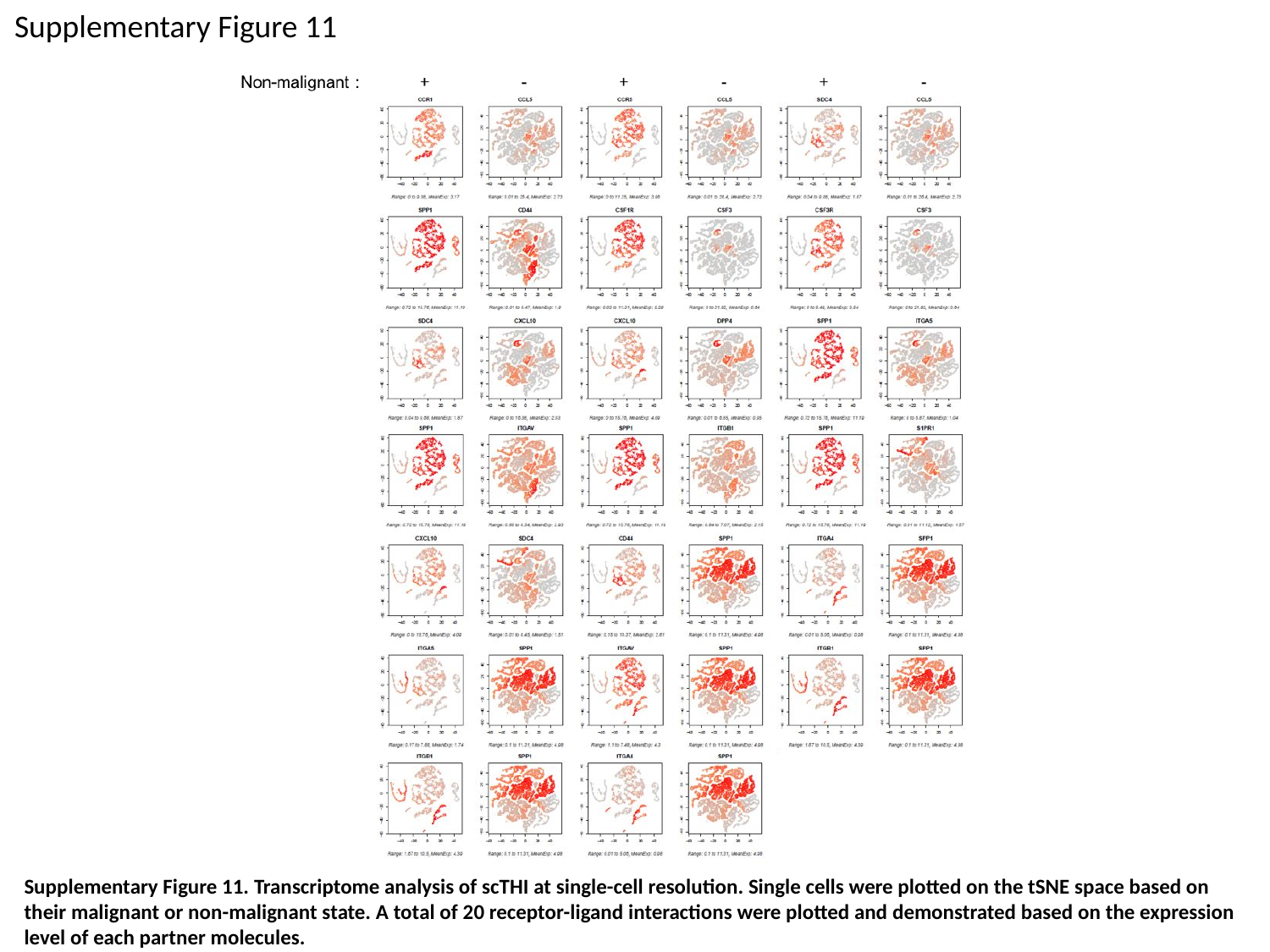

Supplementary Figure 11
Supplementary Figure 11. Transcriptome analysis of scTHI at single-cell resolution. Single cells were plotted on the tSNE space based on their malignant or non-malignant state. A total of 20 receptor-ligand interactions were plotted and demonstrated based on the expression level of each partner molecules.

## Slide 12
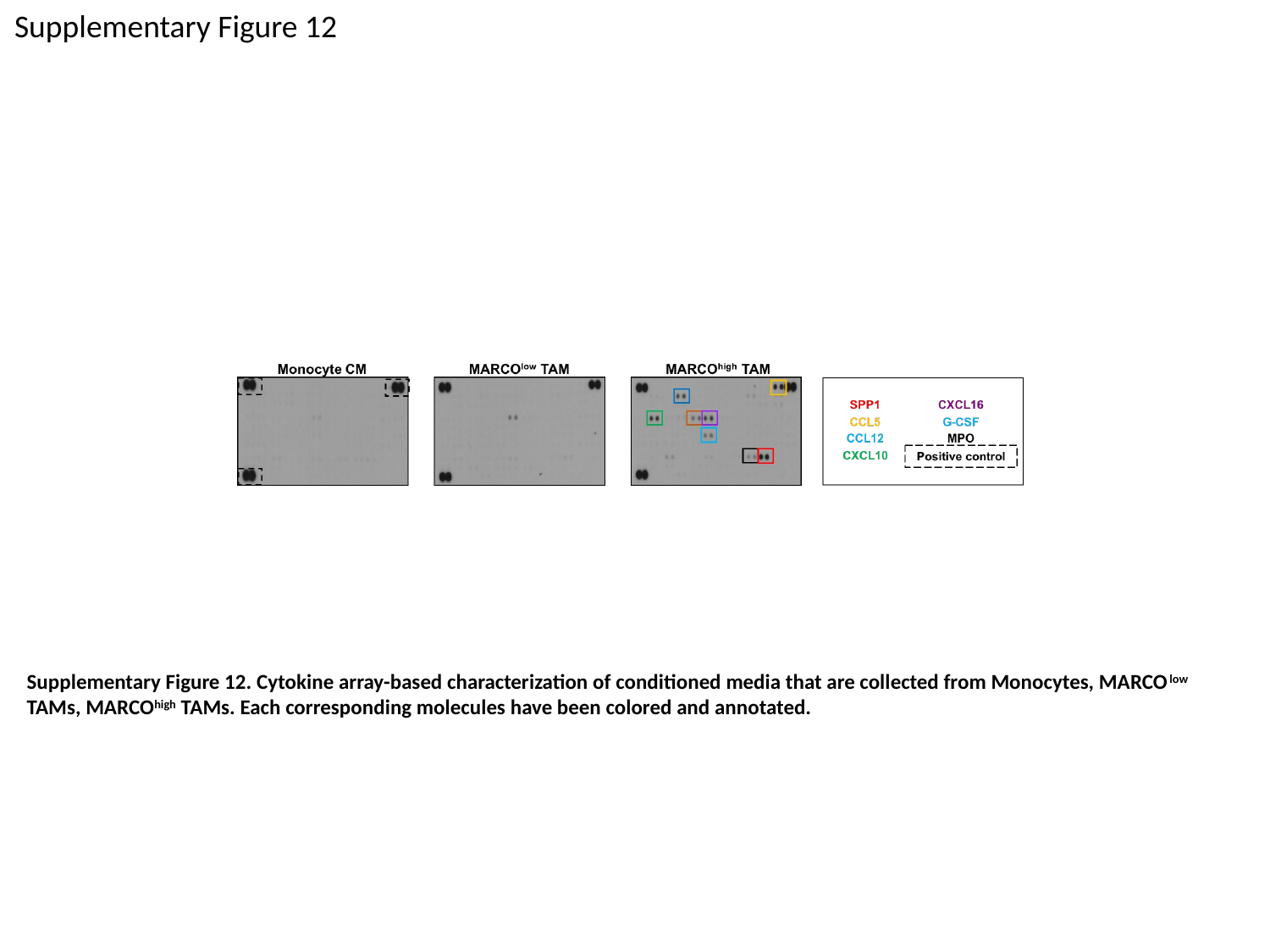

Supplementary Figure 12
Supplementary Figure 12. Cytokine array-based characterization of conditioned media that are collected from Monocytes, MARCOlow TAMs, MARCOhigh TAMs. Each corresponding molecules have been colored and annotated.

## Slide 13
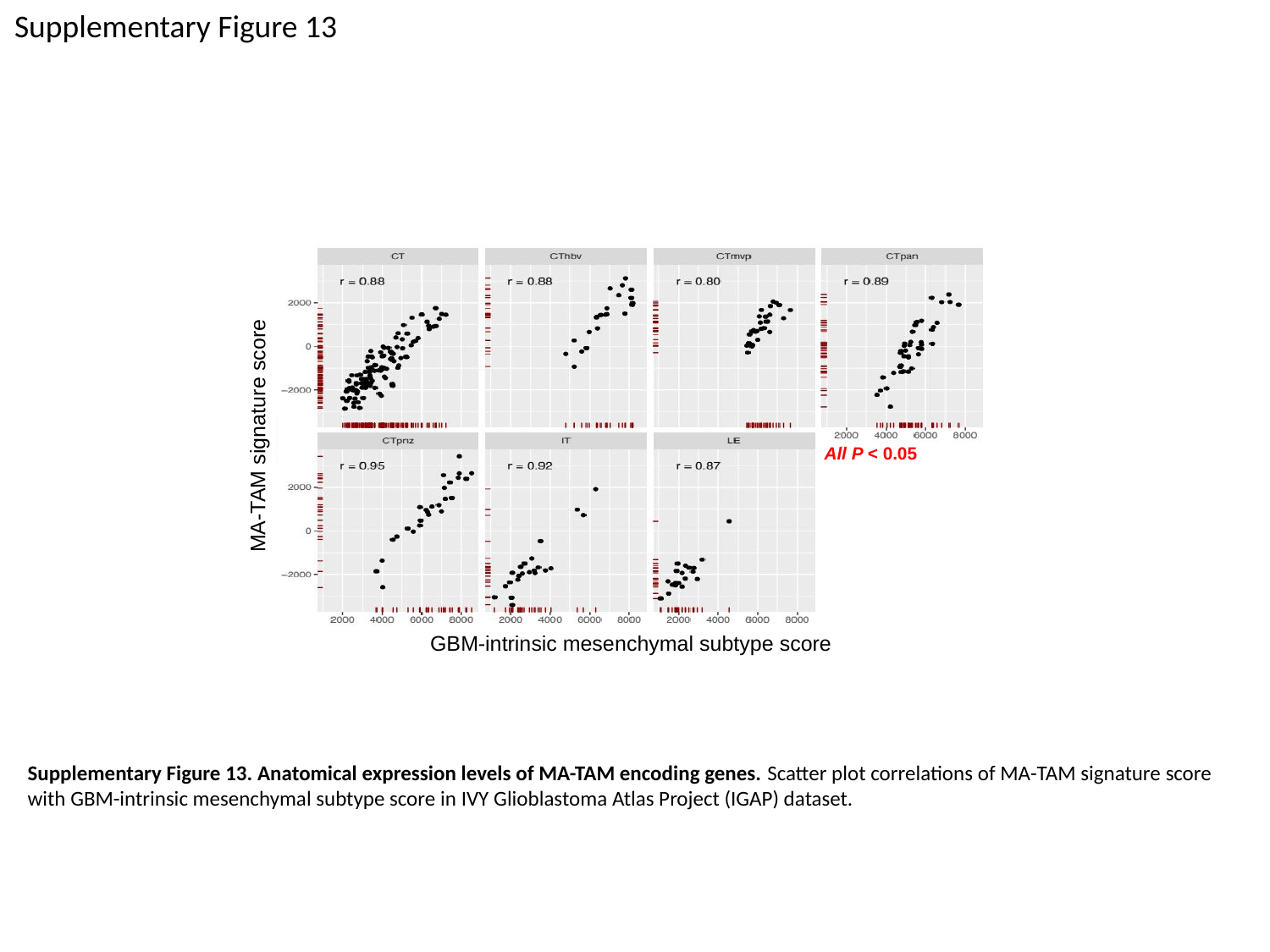

Supplementary Figure 13
MA-TAM signature score
GBM-intrinsic mesenchymal subtype score
All P < 0.05
Supplementary Figure 13. Anatomical expression levels of MA-TAM encoding genes. Scatter plot correlations of MA-TAM signature score with GBM-intrinsic mesenchymal subtype score in IVY Glioblastoma Atlas Project (IGAP) dataset.
