# Supplementary material for: Transcriptional regulatory networks of tumor-associated macrophages that drive malignancy in mesenchymal glioblastoma
Source: Genome Biol. 2020 Aug 26;21:216. doi: 10.1186/s13059-020-02140-x (PMC7448990; doi:10.1186/s13059-020-02140-x)
Supplement: Supplementary file 2 — Additional file 2. Review History. [file 13059_2020_2140_MOESM2_ESM.docx]

Review History

**First round of review**

**Reviewer 1**

**Are you able to assess all statistics in the manuscript, including the appropriateness of statistical tests used?**

Yes, and I have assessed the statistics in my report.

**Comments to author:**

Sa et al. profiled CD45+/CD11b+ and CD45 depleted cells via RNA-seq, sorted from apparently 9 GBMs. They model mesenchymal associated TAMs by over-expressing Pparg in peritoneal macrophages. Supernatants from mutant cultures promote invasion, prolifferation, and angiogenesis in vitro. Co-injection of mutant TAMs promotes malignancy in vivo and enhanced radiation resistance.

The results are novel and of interest to the readership of Genome Biology, since they show how tumor-immune interactions can be identified through a systemic understanding of intra-tumor heterogeneity. The results are generally supported by the data. I would ask the authors to consider the following:

1. The sample size of 9 GBMs is somewhat small. Can your results be confirmed or enhanced by comparing macrophage signatures between mesenchymal-content high and low tumors in the copious public single-cell RNA-seq data for GBM (including from the senior authors)?
2. The authors have made only a limited effort to infer or validate the secreted factors by MARCO-high cells which mediate the phenotypes observed with CM treatment. Receptor-ligand analysis of scRNA-seq, restricted to mesenchymal cells or samples would be informative. Any further validation of neoplastic-macrophage interactions would greatly enhance the manuscript.
3.  It is unclear what the role of MARCO itself is.
4. The transduction of Pparg in macrophages is not reported in the methods. It is not clear how transduced cells were selected, if at all. There is no control for the transduction process in the downstream assays, such as transduction of GFP or an empty vector. Does transfection activate the macropahges, toward M2?
5. A demonstration of targeting the MI-TAM to mesenchymal-cell paracrine signaling network for therapeutic benefit would greatly enhance the manuscript.

Minor:
1. The authors may consider phrasing the original statement of "identifying mesenchymal-inducing macrophages..." as a hypothesis test, or otherwise describe them as "mesenchymal-associated" macrophages or other, as one doesn't know a priori that the mesenchymal phenotype is inducible (let alone by macrophages). Even considering the evidence in the manuscript it may be that certain macrophage-secreted factors select for a mesenchymal phenotype from among the implanted cells rather than induce the mesenchymal phenotype per se.
2. The reporting of sample sizes could be more clear throughout, i.e. human specimens, mice and replicates, etc... at least in the methods.

**Reviewer 2**

**Are you able to assess all statistics in the manuscript, including the appropriateness of statistical tests used?**

No, I do not feel adequately qualified to assess the statistics.

**Comments to author:**

In this manuscript, the authors are trying to investigate the transcriptional regulatory networks in mesenchymal-inducing tumor-associated macrophages (MI-TAMs), which may contribute the malignant phenotypic state of GBM. They identified macrophage receptor with collagenous structure (MARCO) as the most highly differentially expressed gene in mesenchymal GBM associated macrophages comparing with the non-mesenchymal GBM-associated macrophages by whole-transcriptome sequencing. In vitro, they found MARCO high TAMs induced a phenotypic shift towards a mesenchymal cellular state of glioma stem cells, promoting both invasive and proliferative activity, as well as therapeutic resistance to irradiation. In vivo, they showed that MARCOhigh TAMs also significantly accelerated tumor engraftment and growth by co-injection of non-mesenchymal GSCs with the MARCOhigh TAMs. Moreover, they reported that both the MI-TAM master regulators and their target genes were significantly correlated with a poor clinical outcome, and were often associated with genomic aberrations in Nf-1 and PI3K/mTOR/Akt pathway-related genes. Furthermore, they showed that the peripheral blood origin of MI-TAMs, as well as their potential association with a tumor-induced polarization state and an immunosuppressive environment. In general, the idea for this research is interesting, however, this manuscript suffered from several major issues; for instance, some experiments miss rigorous controls, the interpretation of several results is not well supported by the data, and the materials and methods miss critical information (e.g., the culture condition of TAMs, how MARCOhigh TAMs and MARCOkow TAMs were isolated, et al.)

1.     For the Fig 1A and B, the authors identified the MI-TAM signature genes and their transcriptional master regulators using the whole-transcriptome sequencing and coupled with publicly available datasets. It was very hard to understand how these genes were identified. Intermediate steps were not described. Fig. 1A, the scale for different colors indicating the different gene expression are required.
2.     In Fig. 1D, the resolution is too low. The single-color staining pictures should be included. Also, it was quite surprising to see most of the markers in 8 individual non-mesenchymal tumors are totally negative. There was not even background in the non-mesenchymal tumors? This data really makes me question the rigor of this study.
3.     In Figure 2D, E. the experiment should be repeated in a third patient; results are very variable. In Figure 2H, the lable of y scale is confusing. Also how are stem cells defined?
4.     In Fig 3A, co-injection experiment is interesting, however, it also draws many concerns. First, it was not clear in the Materials and Methods how the experiment was actually performed? Mixed GSCs with the TAM then injection, or injected them separately? Do the TAM or Monocytes actually survive in the mouse brain after injection? TAM marker staining should be included in brain tumor samples in the Fig. 3. If the MARCO play an important role in TAM in Non-Mesenchymal GSC to Mesenchymal GSC, blocking MARCO with MARCO agonist or antibody should diminish this effect.
5.     Finally, the authors showed that MARCOhigh-TAM could induce Non-Mesenchymal GSC to Mesenchymal GSC, but Fig. 4A shows that there are still MARCOlow mesenchymal GBM, so how do they maintain their mesenchymal state? Lastly, Fig. 4A shows that even if you block MARCO successfully in human patients (that is a big IF), you probably would only see effect of extending survival time for a couple of weeks. I really don't think this approach is going to benefit patients significantly.

**Authors Response**

**Point-by-point responses to the reviewers’ comments:**

**Response to Reviewer #1**

Major Point 1: The sample size of 9 GBMs is somewhat small. Can your results be confirmed or enhanced by comparing macrophage signatures between mesenchymal-content high and low tumors in the copious public single-cell RNA-seq data for GBM (including from the senior authors)?

*Response: We appreciate the reviewer’s suggestion and acknowledge the limitation on our sample size. Therefore, as the reviewer has suggested, we have analyzed the macrophage signatures between mesenchymal-content high and low tumors at single-cell resolution using single-cell RNA-seq data that are publicly available. We first identified mesenchymal-content high tumors based on the fraction of tumor cells that were classified as mesenchymal in a given tumor. As a result, both PJ017 and PJ032 demonstrated the highest percentage of tumor cells that were classified as mesenchymal (Figure R1A-B). Next, we extracted a group of non-malignant cells from each tumor and investigated expression levels of both MARCO and MA-TAM signature scores (Figure R1C). Notably, macrophages that derived from the mesenchymal-content high tumors (PJ107 and PJ032) demonstrated enrichments of both MARCO and MA-TAM signature score, while mesenchymal-content low tumors (PJ025 and PJ030) exhibited low expression levels, further consolidating our previous findings (Figure R1C).*

**Figure R1**: **Transcriptome analysis of MA-TAM signature and *MARCO* expression at single-cell resolution. (A)** t-SNE analysis of single-cell transcriptomes based on patient (left panel) and GBM subtype (right panel) in malignant tumor cells. **(B)** Single cell fraction of mesenchymal, classical, and proneural tumor cells based on individual patient. **(C)** Patient-level distribution of non-malignant cells on the tSNE space (left panel), expression of *MARCO* (middle left panel), MA-TAM signature enrichment (middle right panel), and boxplot representation of MA-TAM signature enrichments in individual patients.

Major Point 2: The authors have made only a limited effort to infer or validate the secreted factors by MARCO-high cells which mediate the phenotypes observed with CM treatment. Receptor-ligand analysis of scRNA-seq, restricted to mesenchymal cells or samples would be informative. Any further validation of neoplastic-macrophage interactions would greatly enhance the manuscript.

*Response: We thank the reviewer for the critical suggestion and conducted additional analyses and experiments to address the comment. First, we performed receptor-ligand analysis from single-cell RNA-seq data and investigated potential secreted factors by MARCO-high cells. We first distinguished tumor cells based on their expression subtypes and selected those who are identified as mesenchymal. Then, we extracted a group of non-malignant cells with high expression of MARCO. Lastly, we performed scTHI (single cell Tumor-Host Interaction) to identify significantly activated ligand-receptor interactions. As a result, we have detected a total of 598 interactive pairs occurring between MARCO-positive macrophages (Partner A) and Mesenchymal tumor cells (Partner B) and among them 20 interactions involved in secreted factor encoding molecules. These interactions were CCR1-CCL5, CCR5-CCL5, SDC4-CCL5, SPP1-CD44, CSF1R-CSF3, CSF3R-CSF3, SDC4-CXCL10, CXCL10-DPP4, SPP1-ITGA5, SPP1-ITGAV and so forth (Figure R2).*

**Figure R2**: **Transcriptome analysis of scTHI at single-cell resolution.** Single cells were plotted on the tSNE space based on their malignant or non-malignant state. A total of 20 receptor-ligand interactions were plotted and demonstrated based on the expression level of each partner molecules.

*Furthermore, we have conducted cytokine array-based characterization of conditioned media that were derived from either Monocyte, MARCOlow TAMs, or MARCOhigh TAMs. Interestingly, high expression levels of SPP1, CCL5, CCL12, CXCL10, CXCL16, G-CSF, and MPO were identified as MARCOhigh TAM-specific cytokines and SPP1, CCL5, CXCL10, and G-CSF (CSF3) were also identified among significantly activated ligand-receptor interactions, further advocating our findings (Figure R3).*

**Figure R3**: Cytokine array-based characterization of conditioned media that are collected from Monocytes, MARCO^low^ TAMs, or MARCO^high^ TAMs.

Major Point 3: It is unclear what the role of MARCO itself is.

*Response: We sincerely apologize for not providing detailed information on the role of MARCO. MARCO is a class A scavenger receptor that has been involved in essential cellular programs, including phagocytosis, inflammation, etc. Recent reports have recognized MARCO as a potential therapeutic target that is overexpressed and involved in tumor microenvironment (TME) composition and promote poor prognosis across multiple cancer types. Furthermore, expression level of MARCO could be up-regulated through activation of TGF-β and IL-10, which subsequently generates immunosuppressive TME. In that respect, our results demonstrate MARCO as a significant contributor to pro-tumorigenic state of GBM, specifically driving mesenchymal transformation. We suspect that MARCO interacts with a variety of different molecules, including SPP1, CCL5, CCL12 to drive aggressive cellular state of glioblastoma and generates immuno-suppressive environment and targeting of MARCO could potentially prevent such transition. We hypothesize that infiltration of MARCO+ TAM subpopulation could serve as a predictive biomarker for patient prognosis and potential candidate for immunotherapy.*

Major Point 4: The transduction of Pparg in macrophages is not reported in the methods. It is not clear how transduced cells were selected, if at all. There is no control for the transduction process in the downstream assays, such as transduction of GFP or an empty vector. Does transfection activate the macropahges, toward M2?

*Response: We sincerely apologize for not being explicit in providing information on the experimental procedure. We have included the following information in the Methods section.*

*“Overexpression constructs were generated into lentivirus. Briefly, 3-4 x 106 293T cells were seeded in 100-mm culture dishes for 24 hours before co-transfection with 4.5 µg of lentivirus construct (pHRST-IRES-Pparg), 3 µg of psPAX2 (Paired box gene 2), and 1.5 µg of pMD2.G using 27 µl of Lipofectamine 2000. The media were changed after 6 hours and the supernatant containing the lentivirus was harvested 48 hours after the transfection. Viral particles were concentrated and purified using a Lenti-X-concnetrator. Cells were infected with lentivirus in the presence of 6 µg/ml polybrene. eGFP was used as a fluorescent marker to distinguish successfully infected populations.”*

*Furthermore, we have used the same empty construct as control for all our experiments. We apologize for leaving out this information and have corrected it in the manuscript. Lastly, when we transfected macrophages with Pparg-overexpressing construct, we did observe a minor increase in the M2 markers but not significant.*

Major Point 5: A demonstration of targeting the MI-TAM to mesenchymal-cell paracrine signaling network for therapeutic benefit would greatly enhance the manuscript.

*Response: We greatly appreciate the reviewer’s important suggestion. To show that blocking of MA-TAM encoding molecules such as MARCO could provide therapeutic benefit, we have generated and evaluated in-house developed anti-MARCO antibodies. We have searched for MARCO-specific therapeutic antibody or inhibitor that is commercially available, but we could not identify one. Therefore, we have established and assessed novel human MARCO antibodies that have been developed through panning techniques using synthetic human antibody phage libraries. To determine if they specifically bind to the MARCO domain, we have conducted enzyme-linked immunosorbent assay (ELISA) using recombinant human MARCO protein and bovine serum albumin (BSA) as a negative control and confirmed their binding capabilities. Notably, treatment of anti-MARCO antibodies significantly reduced the expression level of MARCO as well as mesenchymal- and stemness-associated markers such as CD44, NANOG, LIF, and MMP2. Although more in-depth molecular validation is required to firmly suggest the therapeutic benefit of targeting MA-TAM, we believe it certainly warrants further investigation (Figure R4).*

**Figure R4**: Immunoblot and qRT-PCR analysis to determine the effects of anti-MARCO antibodies. Tumor cells were first given MARCO^high^ TAM-derived CM and treated with either IgG or anti-MARCO antibodies.

Minor Point 1: The authors may consider phrasing the original statement of "identifying mesenchymal-inducing macrophages..." as a hypothesis test, or otherwise describe them as "mesenchymal-associated" macrophages or other, as one doesn't know a priori that the mesenchymal phenotype is inducible (let alone by macrophages). Even considering the evidence in the manuscript it may be that certain macrophage-secreted factors select for a mesenchymal phenotype from among the implanted cells rather than induce the mesenchymal phenotype per se.

*Response: We agree and thank the reviewer for the valuable suggestion and rephrased all “mesenchymal-inducing macrophages” to “mesenchymal-associated macrophages” in the revised manuscript.*

Minor Point 2: The reporting of sample sizes could be more clear throughout, i.e. human specimens, mice and replicates, etc... at least in the methods.

*Response: We appreciate the reviewer’s suggestion and included the appropriate information in the methods and figure legends.*

**Response to Reviewer #2**

Major Point 1: For the Fig 1A and B, the authors identified the MI-TAM signature genes and their transcriptional master regulators using the whole-transcriptome sequencing and coupled with publicly available datasets. It was very hard to understand how these genes were identified. Intermediate steps were not described. Fig. 1A, the scale for different colors indicating the different gene expression are required.

*Response: We apologize for the lack of clarity in our presentation in Figure 1A and B. In the revised manuscript, we have included a step-wise flowchart on how we identified and determined the MA-TAM (Mesenchymal-Associated Tumor-Associated Macrophages, “we have changed the term based on reviewer #1’s suggestion”) signature genes and their transcriptional master regulators (Figure R5). Furthermore, we sincerely apologize for the absence of the color scale and included it in the revised manuscript.*

**Figure R5**: A schematic illustration on the workflow of identifying MA-TAM specific signature and their master regulators.

**Major** Point 2: In Fig. 1D, the resolution is too low. The single-color staining pictures should be included. Also, it was quite surprising to see most of the markers in 8 individual non-mesenchymal tumors are totally negative. There was not even background in the non-mesenchymal tumors? This data really makes me question the rigor of this study.

*Response: We apologize for the lack of high-resolution images. In the revised manuscript, we have included single-color staining pictures for each respective marker (Figure R6). However, we could not obtain immunofluorescence-based images for single color staining due to the limited export option in the software (InForm), therefore, all single-color pictures are provided in immunohistochemical images. Furthermore, we have thoroughly reanalyzed the non-mesenchymal tumors and unfortunately, we did not identify any cells that positively expressed for these MA-TAM markers. We will be happy to provide all stained images if requested.*

**Figure R6**: Representative immunohistochemical images of MA-TAM markers, including BATF, CD68, MARCO, PPARy, SPI1, and YKL40.

**Major Point** 3: In Figure 2D, E. the experiment should be repeated in a third patient; results are very variable. In Figure 2H, the lable of y scale is confusing. Also how are stem cells defined?

*Response: We thank the reviewer’s important suggestion and performed additional experiments using a third patient-derived tumor cells. Consistent with our previous results, treatment of MARCO and CCL7 recombinant proteins significantly elevated the mesenchymal markers, including CD44, NANOG, LIF, and MMP-2 mRNA expression levels and CD44, TAZ, and NANOG protein expression levels (Figure R7).*

**Figure R7**: **(A)** qRT-PCR analysis to determine the effects of MARCO and CCL7 on the expression of mesenchymal, stemness and cellular invasion markers, including CD44, NANOG, LIF, and MMP-2 (left panel). **(B)** Immunoblot analysis of CD44, TAZ, and NANOG activity in GSC treated with control, MARCO, or CCL7 recombinant proteins. Vinculin was used as a loading control.

*Furthermore, we apologize for not providing detailed explanation of the y-scale in Figure 2H. Stem cell frequency is determined by web-based tool “ELDA” (extreme limiting dilution analysis,* [*http://bioinf.wehi.edu.au/software/elda/)*](http://bioinf.wehi.edu.au/software/elda/)) *based on the number of spheres that have been observed in a given well. We have included the additional information in the methods section in the newly revised manuscript.*

*“For ex vivo analysis of GSCs co-injected with monocytes, MARCOlow TAMs, or MARCOhigh TAMs, PDX-derived tumors from each group were freshly dissociated into single cells and seeded in 96-well plates at 1–100 cells/well. Cells were treated with 5 Gy ionizing radiation using the IBL 437C blood irradiator (CIS Bio International, Saclay, France). After 2 weeks, each well was examined for neurosphere formation. The LDA clonogenic index is calculated as the inverse of the x-intercept of the regression between the number of wells without spheres and the number of cells seeded. The frequency of stem-like clonogenic cells was determined using Extreme Limiting Dilution Analysis (*[*http://bioinf.wehi.edu.au/software/elda/)*](http://bioinf.wehi.edu.au/software/elda/))*.”*

Major Point 4: In Fig 3A, co-injection experiment is interesting, however, it also draws many concerns. First, it was not clear in the Materials and Methods how the experiment was actually performed? Mixed GSCs with the TAM then injection, or injected them separately? Do the TAM or Monocytes actually survive in the mouse brain after injection? TAM marker staining should be included in brain tumor samples in the Fig. 3. If the MARCO play an important role in TAM in Non-Mesenchymal GSC to Mesenchymal GSC, blocking MARCO with MARCO agonist or antibody should diminish this effect.

*Response: We sincerely apologize for the lack of detailed information on the experimental procedure and thank the reviewer for pointing out this mistake. The experiments were performed by mixing GSCs with monocytes or TAMs and co-injected together. Furthermore, as the reviewer has suggested, we performed immune-histochemical analysis of TAM markers and discovered that tumors that were co-injected with MARCOhigh TAMs demonstrated significant infiltration of M2 macrophages, shown by high expression of CD163 and CD68 (Figure R8), which promoted mesenchymal transformation of adjacent tumor cells and shortened survival span of in vivo models (Main Figure 3A-C).*

**Figure R8**: **Representative immunohistochemical images of M2 macrophage markers, including CD163 and CD68 in mouse models.**

*Furthermore, in order to determine if blocking MARCO with MARCO antibody could potentially diminish the effect of MA-TAMs in mesenchymal transformation, we have generated and evaluated an in-house developed anti-MARCO antibody. We have searched for MARCO-specific therapeutic antibody or inhibitor that is commercially available, but we could not identify one. Therefore, we have established and assessed novel human MARCO antibodies that have been developed through panning techniques using synthetic human antibody phage libraries. To determine if they specifically bind to the MARCO domain, we have conducted enzyme-linked immunosorbent assay (ELISA) using recombinant human MARCO protein and bovine serum albumin (BSA) as a negative control and confirmed their binding capabilities. Notably, treatment of anti-MARCO antibodies significantly reduced the expression level of MARCO as well as mesenchymal- and stemness-associated markers such as CD44, NANOG, LIF, and MMP2. Although more in-depth molecular validation is required to firmly suggest the therapeutic benefit of targeting MA-TAM, we believe it certainly warrants further investigation (Figure R9).*

**Figure R9**: Immunoblot and qRT-PCR analysis to determine the effects of anti-MARCO antibodies. Tumor cells were first given MARCO^high^ TAM-derived CM and treated with either IgG or anti-MARCO antibodies.

Major Point 5: Finally, the authors showed that MARCOhigh-TAM could induce Non-Mesenchymal GSC to Mesenchymal GSC, but Fig. 4A shows that there are still MARCOlow mesenchymal GBM, so how do they maintain their mesenchymal state? Lastly, Fig. 4A shows that even if you block MARCO successfully in human patients (that is a big IF), you probably would only see effect of extending survival time for a couple of weeks. I really don't think this approach is going to benefit patients significantly.

*Response: We greatly appreciate the reviewer’s insightful observation. We have previously identified a set of essential molecules, besides MARCO from mesenchymal-associated tumor associated macrophages that promote mesenchymal transformation in GBM. Therefore, we suspect that there is a subset of tumors who can maintain their mesenchymal cellular state through activation of different mesenchymal-associated genes, such as CCL7, CD180, MMP8, CR1, etc. and their potential significance warrants further investigation. Lastly, we do acknowledge and agree with the reviewer that inhibition of MARCO alone may only provide limited survival benefits. However, we propose that combination of anti-MARCO treatment with immune-checkpoint inhibitors such as PD-1, PD-L1, or CTLA4 could significantly enhance the therapeutic effects of immunotherapy in GBM patients. Consistently, recent studies have shown empirical evidence in the role of pro-tumorigenic tumor-associated macrophages during immunotherapy and we propose combinational treatment of targeting TAMs via anti-MARCO treatment with immune checkpoint blockades could potentially provide therapeutic benefits in GBM patients[1, 2].*

*References*

*1.	de Groot J, Penas-Prado M, Alfaro-Munoz K, Hunter K, Pei BL, O'Brien B, Weathers SP, Loghin M, Kamiya Matsouka C, Yung WKA, et al: Window-of-opportunity clinical trial of pembrolizumab in patients with recurrent glioblastoma reveals predominance of immune-suppressive macrophages. Neuro Oncol 2020, 22:539-549.*

*2.	Aslan K, Turco V, Blobner J, Sonner JK, Liuzzi AR, Nunez NG, De Feo D, Kickingereder P, Fischer M, Green E, et al: Heterogeneity of response to immune checkpoint blockade in hypermutated experimental gliomas. Nat Commun 2020, 11:931.*

**Second round of review**

**Reviewer 1**

The authors have addressed my concerns and responded adequately to the other reviewer.

**Reviewer 2**

The authors have addressed all my concerns.
